# Uncoupling of Mitosis and Cytokinesis Upon a Prolonged Arrest in Metaphase Is Influenced by Protein Phosphatases and Mitotic Transcription in Fission Yeast

**DOI:** 10.3389/fcell.2022.876810

**Published:** 2022-07-18

**Authors:** Nathalia Chica, Marina Portantier, Mari Nyquist-Andersen, Silvia Espada-Burriel, Sandra Lopez-Aviles

**Affiliations:** ^1^ Centre for Molecular Medicine Norway (NCMM), Nordic EMBL partnership, Faculty of Medicine, University of Oslo, Oslo, Norway; ^2^ Institute of Biosciences (IBV), Faculty of Mathematics and Natural Sciences, University of Oslo, Oslo, Norway

**Keywords:** metaphase arrest, APC/C^Slp1^, phosphatases, PP2A-B55, PP2A-B56, CDK, cytokinesis, fission yeast

## Abstract

Depletion of the Anaphase-Promoting Complex/Cyclosome (APC/C) activator Cdc20 arrests cells in metaphase with high levels of the mitotic cyclin (Cyclin B) and the Separase inhibitor Securin. In mammalian cells this arrest has been exploited for the treatment of cancer with drugs that engage the spindle assembly checkpoint and, recently, with chemical inhibitors of the APC/C. While most cells arrested in mitosis for prolonged periods undergo apoptosis, others skip cytokinesis and enter G1 with unsegregated chromosomes. This process, known as mitotic slippage, generates aneuploidy and increases genomic instability in the cancer cell. Here, we analyze the behavior of fission yeast cells arrested in mitosis through the transcriptional silencing of the Cdc20 homolog *slp1*. While depletion of *slp1* readily halts cells in metaphase, this arrest is only transient and a majority of cells eventually undergo cytokinesis and show steady mitotic dephosphorylation. Notably, this occurs in the absence of Cyclin B (Cdc13) degradation. We investigate the involvement of phosphatase activity in these events and demonstrate that PP2A-B55^Pab1^ is required to prevent septation and, during the arrest, its CDK-mediated inhibition facilitates the induction of cytokinesis. In contrast, deletion of PP2A-B56^Par1^ completely abrogates septation. We show that this effect is partly due to this mutant entering mitosis with reduced CDK activity. Interestingly, both PP2A-B55^Pab1^ and PP2A-B56^Par1^, as well as Clp1 (the homolog of the budding yeast mitotic phosphatase Cdc14) are required for the dephosphorylation of mitotic substrates during the escape. Finally, we show that the mitotic transcriptional wave controlled by the RFX transcription factor Sak1 facilitates the induction of cytokinesis and also requires the activity of PP2A-B56^Par1^ in a mechanism independent of CDK.

## Introduction

The most fundamental goal of the cell cycle, the passing on of genetic material from one generation to the next, requires an exquisite control of S phase, mitosis and cytokinesis. Ordered completion of these events is the consequence of the concerted action of cyclin-dependent kinases (CDKs) and CDK-counteracting phosphatases, as well as ubiquitin ligases that bring about the degradation of key cell cycle regulators once their functions have been fulfilled. During the metaphase to anaphase transition, the Anaphase Promoting Complex/Cyclosome (APC/C), together with its activator Cdc20, coordinates chromosome segregation with CDK downregulation, thus driving the cell out of mitosis.

From the systems-level perspective, mitosis can be divided into a first period of rising mitotic CDK activity and low APC/C activity, and a second period that is governed by high APC/C activity. During the first period (prophase-metaphase), chromosomes condense, the mitotic spindle forms, the nuclear envelope breaks down, and mitotic CDK substrates become extensively phosphorylated. Once all chromosomes are correctly bioriented in the metaphase spindle, satisfaction of the mitotic checkpoint (AKA spindle assembly checkpoint or SAC) releases the APC/C activator Cdc20. APC/C^Cdc20^ mediated degradation of the Separase inhibitor Securin and of the mitotic cyclin (Cyclin B) results in chromosome segregation and the downregulation of CDK activity, respectively (Morgan, 2007). However, inactivation of CDK on its own is not sufficient to revert the phosphorylation events that drove the cell into mitosis, and CDK-counteracting phosphatases become essential to lead the cell out of mitosis and into G1 (Visintin et al., 1998; Queralt et al., 2006; Skoufias et al., 2007; López-Avilés et al., 2009; Mochida et al., 2009; Manchado et al., 2010; Bouchoux and Uhlmann, 2011; Touati et al., 2019).

Activation of the mitotic checkpoint is a common strategy in the treatment of cancer. Drugs that hinder microtubule polymerization (e.g., nocodazole and vinca alkaloids), that prevent their dynamic depolymerization (e.g., taxol) or that inhibit the activity of motor proteins (e.g., inhibitors of the plus end-directed kinesin Eg5, required for spindle pole separation) all promote a checkpoint response that results in cell cycle arrest in mitosis with high mitotic CDK activity. Cells arrested in mitosis for prolonged periods eventually undergo apoptosis but, in some instances, they can slip from the arrest and exit mitosis without dividing, a process known as mitotic slippage (reviewed in Topham and Taylor, 2013). Whether a cell undergoes one fate or the other seems to depend on the balance between the level of CDK activity and the presence of pro-apoptotic signals. Thus, if CDK activity drops below a certain threshold before apoptosis has been effectively triggered, the cell will exit mitosis and enter G1. Steady Cyclin B degradation during the arrest is thought to be the main cause of mitotic slippage (Brito and Rieder, 2006; Lee et al., 2010; Sloss et al., 2016), although Wee1-mediated inactivation of Cyclin B-CDK1 through Tyr15 phosphorylation is also thought to contribute to this phenotype (Visconti et al., 2015). Cells that skip cytokinesis become aneuploid and will either undergo apoptosis in G1, become senescent or continue dividing as genomically unstable cells. This last fate contributes to the CIN phenotype often observed in cancer cells (Hanahan and Weinberg, 2011).

While molecules that disrupt microtubule dynamics are very useful anti-cancer drugs, their efficacy depends on the presence of a fully functional mitotic checkpoint. Moreover, since they do not only disrupt microtubules in mitosis but also affect the cytoskeleton of interphase cells, their use leads to significant side effects.

An alternative to these drugs is the direct inhibition of the APC/C. Deletion of *CDC20* in mouse MEFs was shown to stably arrest cells in mitosis (Manchado et al., 2010) and a combination of drugs targeting the APC/C (Apcin and ProTAME) could slow down mitotic exit in RPE1 cells (Sackton et al., 2014).

Similarly, in budding yeast transcriptional silencing of *CDC20* (e.g., using a *GAL-CDC20* allele) uniformly arrests cells in metaphase with high CDK activity and mitotic phosphorylation.

In fission yeast, *nda3* mutants [encoding beta-tubulin (Umesono et al., 1983; Hiraoka et al., 1984)] are commonly used to arrest cells in mitosis and overexpression of the mitotic checkpoint proteins Mad2 or Mph1 imposes a cell cycle arrest in metaphase and prevents cytokinesis (He et al., 1997, 1998). Nevertheless, mutants of several subunits of the APC/C depict the so-called *cut* (cell untimely torn) phenotype (Hirano et al., 1986; Samejima et al., 1993; Yamashita et al., 1996, 1999; Yamada et al., 1997; reviewed in Yanagida et al., 1999), where cytokinesis proceeds regardless of the fact that chromosomes failed to segregate and decondense.

In fission yeast, cytokinesis is governed by the septation initiation network (SIN), analogous to budding yeast mitotic exit network (MEN) and similar to the metazoan Hippo pathway. The SIN pathway comprises a small GTPase [Spg1 (Schmidt et al., 1997)], its bipartite GAP [Byr4:Cdc16 (Furge et al., 1998)], the SIN activator Etd1 (Daga et al., 2005), and a cascade of three protein kinases that function downstream of Spg1: Cdc7, Sid1:Cdc14 and Sid2:Mob1 [reviewed in Krapp and Simanis (2008), Johnson et al. (2012)]. SIN signalling occurs at the spindle pole body (SPB), where these proteins are tethered via the scaffold formed by Cdc11 and Sid4. During interphase, Spg1 is kept in its GDP-bound form through the interaction with Byr4:Cdc16. As the cell enters mitosis, this interaction is dissolved, allowing Spg1 activation and subsequent recruitment of Cdc7. Inactivation of Byr4 is brought about by the kinase Plo1 together with the mitotic CDK complex (Cdc13:Cdc2) through phosphorylation (Johnson and Gould, 2011; Rachfall et al., 2014). However, at anaphase onset, when CDK activity declines, Byr4 and Cdc16 return to the old SPB, resulting in SIN signalling only being triggered at the new SPB (Cerutti and Simanis, 1999). Although mitotic CDK is required for the initial activation of the SIN through Byr4 inactivation, it also prevents the recruitment of the PAK-related kinase complex Sid1:Cdc14, thus precluding SIN signalling (Guertin et al., 2000; Csikász-Nagy et al., 2007). Thus, high CDK licenses the SIN while preventing its firing, so that septation only occurs in anaphase once the mitotic cyclin Cdc13 has been degraded and chromosomes are segregated. Nevertheless, when problems arise during mitosis and the SAC is engaged, the SIN needs to be actively inhibited to preclude cytokinesis. This inhibition is mediated by the E3 ubiquitin ligase Dma1 (*defective in mitotic arrest*) (Murone and Simanis, 1996), which ubiquitinates the SIN scaffold Sid4. In its ubiquitinated form, Plo1 cannot bind Sid4 and hence Byr4 phosphorylation is abolished (Johnson and Gould, 2011).

The fact that, in the absence of Dma1, CDK inhibition of the downstream SIN kinases is not sufficient to prevent cytokinesis during a checkpoint-induced mitotic arrest is in agreement with the *cut* phenotypes observed in mutants of the APC/C [e.g., *slp1, cut4, cut9* (Samejima et al., 1993; Matsumoto, 1997)] or the proteasome [e.g., *mts2* (Yen et al., 2003)]*.* In these mutants, Cdc13 and Securin degradation is prevented without prompting the checkpoint.

This observation also raises the possibility that, while natural oscillations in CDK activity during an unperturbed cell cycle are sufficient to coordinate chromosome segregation and cytokinesis, abnormal upregulation of CDK during a prolonged mitotic arrest might trigger additional mechanisms to exit mitosis.

Here, we characterize the effect of the acute depletion of the *Schizosaccharomyces pombe* APC/C activator Slp1 [the ortholog of budding yeast Cdc20 (Kim et al., 1998)] in the induction and maintenance of a mitotic arrest. In accordance with its essential role in the degradation of Cdc13 and Securin, transcriptional repression of *slp1* halts mitotic progression in metaphase with high levels of Cdc13 and mitotic phosphorylation. However, cytokinesis is only transiently blocked and cells undergo untimely division, leading to mitotic catastrophe or unequal chromosome segregation. We also investigate the involvement of CDK-counteracting phosphatases and the mitotic transcriptional wave in these events and demonstrate that two different PP2A complexes (PP2A-B55^Pab1^ and PP2A-B56^Par1^) play opposite roles in the induction of cytokinesis in our experimental set up. Interestingly, we show that these phosphatases cooperate with the Cdc14-like phosphatase Clp1 in the reversion of mitotic phosphorylation. Finally, we correlate mitotic escape with the expression of the mitotic transcriptional cluster, and show the involvement of PP2A-B56^Par1^ in its regulation.

## Results

### Depletion of *slp1* Arrests Cells in Metaphase but Cannot Prevent Cytokinesis

Successful cell division relies on cytokinesis occurring only once chromosomes have been segregated. In fission yeast this is partly accomplished by the inhibition of Sid1:Cdc14 recruitment to the SPB by CDK (Guertin et al., 2000). Thus, only once CDK activity decays following APC/C^Slp1^ mediated Cdc13 degradation at the metaphase to anaphase transition can cytokinesis take place. However, original screens aimed at identifying proteins involved in the onset and/or progression of sister chromatids separation rendered a number of mutants with *cut* phenotype. Further characterization of the gene products of these alleles showed that many of them encoded conserved subunits of the APC/C [reviewed in Yanagida et al. (1999)]. In these *cut* mutants, Cdc13 degradation is presumably prevented; still, cells undergo cytokinesis prior to anaphase.

**FIGURE 1:**
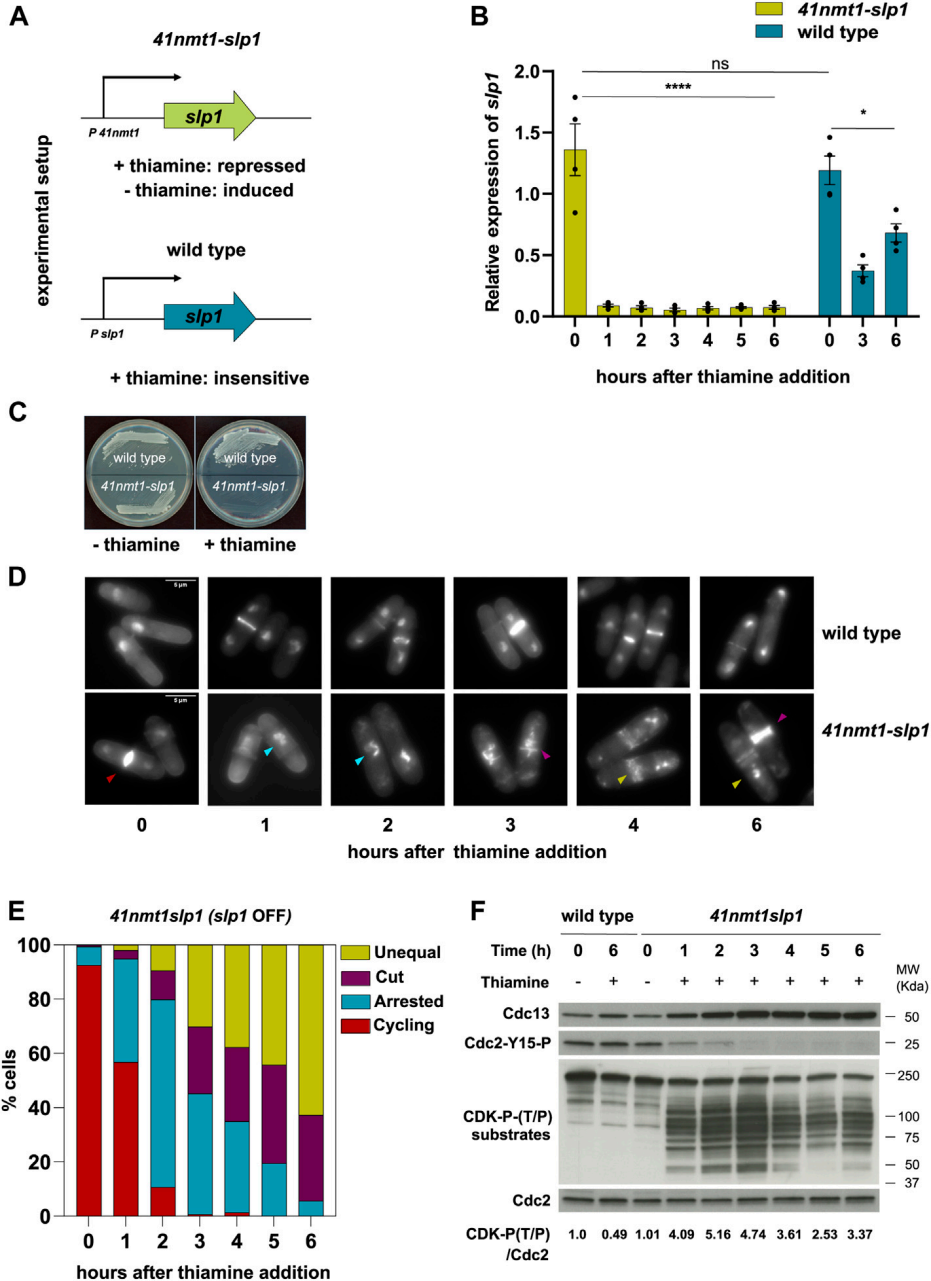
Depletion of *slp1* arrests cells in metaphase but cannot prevent cytokinesis. **(A)** Overview of the experimental setup used to repress the expression of *slp1* under the control of *p41-nmt1*. *Slp1* is repressed in the presence of 15 µM thiamine (+T) and expressed in the absence of thiamine (−T). Wild type cells were used as control for the endogenous expression of *slp1.* **(B,D–F)** cells were grown exponentially in EMM (*p41-nmt1* promoter induced) and shifted to EMM +T (*p41-nmt1* promoter repressed) at 30°C. Samples were collected at the indicated time points for mRNA expression, microscopy, and western blot. **(B)** mRNA expression of *slp1* in wild type and *p41-nmt1:slp1* cells. Expression is relative to actin and was determined by qPCR. Mean and SEM of four biological replicates are shown. Statistical significance was determined by a two-way ANOVA with Tukey’s multiple comparisons test. ns: *p* > 0.05; ****: *p* ≤ 0.0001. **(C)** Image of wild type andp41-*nmt1:slp1* cells streaked on EMM agar plates that contain 15 µM thiamine or not at 30°C. **(D)** Images of ethanol-fixed cells stained with DAPI to visualise DNA compactation and blankophor to visualise cell wall and septum by microscopy. In the lower panels, Differently-colored arrowheads indicate the different phenotypes scored in **(E)**: Cycling cells (red), metaphase-arrested cells with condensed chromosomes (cyan), *cut* cells (purple) and septated cells with unequal segregation of chromosomes (yellow). Scale bar 5 µm. **(E)** The percentage of cycling cells, metaphase-arrested cells, and arrested cells that underwent cytokinesis (cut or unequal) were determined at the indicated time points after the addition of thiamine to the culture. At least 200 cells were counted per time point. **(F)** Cdc13 levels, phosphorylation at T/P sites in CDK substrates and Cdc2 phosphorylated at Y15 served as indicators of CDK activity and were detected by western blot. Cdc2 (PSTAIR) was used as a protein loading control. The numbers under the western blot indicate CDK-substrate Phosphorylation (P-T/P) quantification relative to Cdc2.

It was previously shown that some *cut* mutants represent hypomorphic alleles and that full depletion of their products results in a complete block of Cdc13 degradation and cytokinesis (Chang et al., 2001). To reconcile these observations, we decided to investigate the consequences of the depletion of the APC/C activator Slp1 in the progression of mitosis and cytokinesis. For this aim, we generated a yeast strain were *slp1* was under the control of the thiamine-repressible *p41-nmt1* promoter (Figure 1A). This approach had been previously used to study chromosome dynamics in metaphase-arrested cells (Petrova et al., 2013; Kakui et al., 2017; Hocquet et al., 2018). Addition of thiamine to the growing medium efficiently suppressed the expression of *slp1* (Figure 1B) and, in agreement with Slp1’s essentiality in the cell, prevented colony formation (Figure 1C). Already after 1 h of *slp1* transcriptional repression, over 40% of the cells were arrested in metaphase with condensed chromosomes (Figures 1D,E). After 2 h, the number of arrested cells increased to 90%, but of those, 20% depicted either a *cut* phenotype or had displaced the undivided nucleus to one side of the cell and undergone division, giving rise to septated cells containing an empty compartment (hereafter referred to as unequal segregation). This phenotype worsened over time, and after 6 h of thiamine addition, virtually all cells had undergone septation in the presence of condensed, unsegregated chromosomes (Figures 1D,E). Of note, cell separation did not occur in these cells and often a second septum was placed in close proximity to the first one. Given the role of the mitotic CDK complex Cdc13:Cdc2 in preventing firing of the SIN, we next tested whether leaky expression of *slp1* could result in Cdc13 degradation during the arrest. However, this was not the case, since Cdc13 increased significantly after just 1 h of *slp1* repression and stayed high for the remainder of the experiment (Figure 1F). Interestingly, after 3 h, Cdc13 did not accumulate further as the arrest persisted and proteomic analysis indicated that it reached a plateau in the last time points of the experiment (Supplementary Table S3). Indeed, the values of Cdc13 were comparable to the values of Cdc2, suggesting that sufficient Cdc2 was present in the cell and that Cdc13 detected in our experiments was likely Cdc2-bound.

In addition, inhibitory phosphorylation of Tyr15 of Cdc2, which prevents activation of the mitotic complex during G2 phase and is reverted as cells enter mitosis, followed the opposite dynamic: it decreased after 1 h of thiamine addition and it was completely absent after 3 h of treatment (Figure 1F). Cdc13:Cdc2 establishes a double-negative feedback with Wee1 and a double-positive feedback with Cdc25, the kinase and phosphatase responsible for Tyr15 phosphorylation and dephosphorylation, respectively (Kovelman and Russell, 1996; Aligue et al., 1997; Sveiczer et al., 2000). Therefore, the absence of Tyr15 phosphorylation is an indication of the activity of Cdc13:Cdc2 in our experimental set up. In addition, we analyzed CDK-substrate phosphorylation in whole cell extracts using an antibody that recognizes the phosphorylated CDK consensus P-Thr-Pro. As expected, the signal increased as Cdc13 accumulated in the cell and was maximal after 3 h of *slp1* repression. Surprisingly, the signal was not steady and decayed from that point on, returning to the level achieved after 1 h of treatment (Figure 1F, lower panel). Importantly, septation preceded the decline in substrate dephosphorylation, suggesting that it was not a consequence of a decrease in CDK activity.

All in all, these results indicate that: first, *slp1* silencing efficiently blocks Cdc13 degradation; second, septation can take place in the presence of high CDK activity; finally, some degree of substrate dephosphorylation occurs during a prolonged mitotic arrest.

### Protein Phosphatases Influence the Behavior of the System During a Sustained Mitotic Arrest

Since we were able to detect a loss of CDK-substrate phosphorylation despite the high level of Cdc13 in metaphase arrested cells, we next decided to investigate whether CDK-counteracting phosphatases participate in the observed phenotypes.

In metazoans, phosphatases belonging to the PP2A-B55 family have been shown to be required to revert mitotic phosphorylation (Mochida et al., 2009; Vigneron et al., 2009; Burgess et al., 2010; Manchado et al., 2010). In budding yeast, the phosphatase Cdc14 was shown to be essential to dephosphorylate CDK substrates during mitotic exit (Visintin et al., 1998). Moreover, since those substrates include the CDK inhibitor Sic1 (Visintin et al., 1998) and the APC/C activator Cdh1 (Zachariae et al., 1998), Cdc14 is also instrumental to sustain the downregulation of CDK (López-Avilés et al., 2009). However, recent studies have shown that, also in budding yeast, phosphatases from the PP2A family are required during exit from mitosis (Touati et al., 2019).

In fission yeast, the Cdc14-like phosphatase Clp1 is not essential for mitotic exit (Cueille et al., 2001; Trautmann et al., 2001). However, its loss advances mitotic entry through the regulation of the CDK-activating phosphatase Cdc25 (Esteban et al., 2004; Wolfe and Gould, 2004). In addition, Clp1 lies at the bottom of the SIN and plays a role in the maintenance of the actomyosin ring and in the cytokinesis checkpoint, preventing additional rounds of division after unsuccessful cytokinesis (Mishra et al., 2005; Chen et al., 2008).

PP2A phosphatases are heterotrimeric complexes comprising a scaffolding subunit (A), a regulatory subunit (B) and a catalytic subunit (C) (reviewed in (Janssens et al., 2008)). Regulatory subunits provide substrate specificity as well as control over the spatial and temporal distribution of the complex. In fission yeast, there is only one scaffolding subunit (Paa1) which is essential, two catalytic subunits (Ppa1 and Ppa2) and three B-type regulatory subunits: Pab1 (B or B55), Par1 and Par2 (B′ or B56). Moreover, a non-canonical complex (SIP) containing Paa1, an alternative catalytic subunit (Ppa3) and four additional components (Csc1-4), was implicated in the regulation of the SIN and the generation of asymmetry during septation (Singh et al., 2011). PP2A-B55^Pab1^ is known to regulate mitotic CDK activity by counteracting the CDK-mediated inhibition of Wee1 and activation of Cdc25 (Kinoshita et al., 1993; Chica et al., 2016). Moreover, elegant studies from the Hagan laboratory proved the existence of a phosphatase relay during mitotic exit. According to their model, step-wise activation of PP1, PP2A-B55^Pab1^ and, ultimately, PP2A-B56^Par1^ is required for orderly exit from mitosis in fission yeast (Grallert et al., 2015). In addition, both PP2A-B55^Pab1^ and PP2A-B56^Par1^ have been shown to negatively regulate septation (Jiang and Hallberg, 2000; Le Goff et al., 2001; Krapp et al., 2003; Lahoz et al., 2010; Goyal and Simanis, 2012).

**FIGURE 2:**
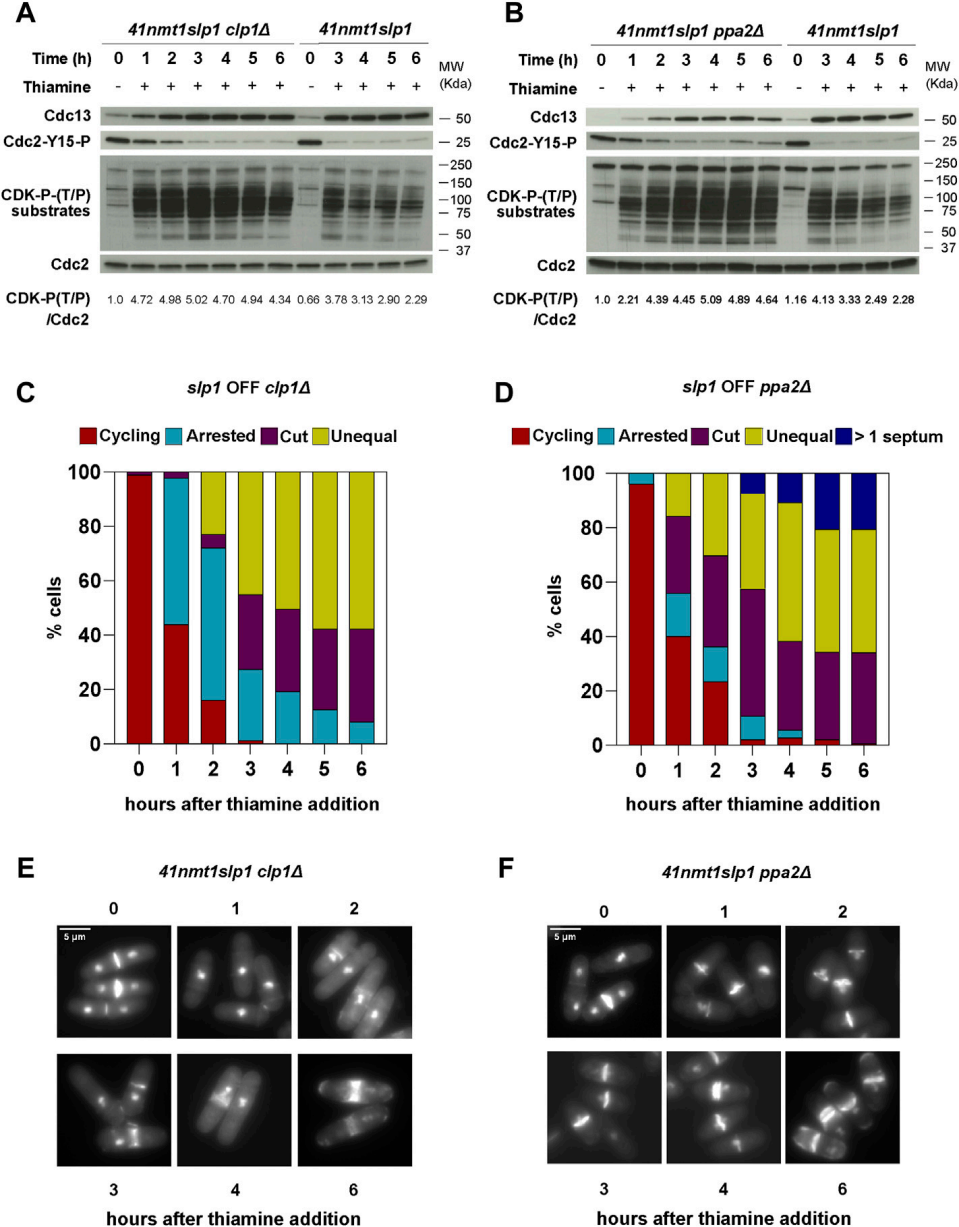
Protein phosphatases influence the mitotic arrest. **(A–F)** cells were grown exponentially in EMM (*p41-nmt1* promoter induced) and shifted to EMM +T (*p41-nmt1* promoter repressed) at 30°C. Samples were collected at the indicated time points for microscopy and western blot. **(A,B)** Cdc13 levels, phosphorylation at T/P sites in CDK substrates and Y15-Cdc2 phosphorylation in *p41-nmt1:slp1* and *p41-nmt1:slp1 clp1Δ* **(A)** or *p41-nmt1:slp1* and *p41-nmt1:slp1 ppa2Δ* strains **(B)** were detected by western blot. Cdc2 (PSTAIR) was used as a protein loading control. The numbers under the western blot indicate CDK-substrate Phosphorylation (P-T/P) quantification relative to Cdc2. **(C,D)** The percentage of cycling cells, metaphase-arrested cells, and arrested cells that underwent cytokinesis (cut or unequal) were determined at the indicated time points after the addition of thiamine to the culture. At least 200 cells were counted per time point. **(E,F)** Representative images of mitotic features distributions from DAPI and Blankophor-stained ethanol-fixed cells in **(C,D)**. Scale bar 5 µm.

In agreement with the role of Clp1 preventing premature mitotic entry, depletion of *slp1* in the *clp1*Δ background led to premature and enhanced CDK-substrate phosphorylation (Figure 2A). In addition, we could observe a higher proportion of cells arrested in metaphase after just 1 h following thiamine addition (Figures 2A,C). CDK-substrate phosphorylation was more prominent than in the wild type control but it was not sustained and started to decrease after 3 h of arrest. Deletion of *ppa2* also advanced mitotic phosphorylation of CDK substrates but, in contrast to *clp1*Δ, phosphorylation was maintained throughout the time course (Figure 2B). In both cases, Cdc13 behaved as in the wild type background, and Cdc2-Tyr15 remained low, only showing a slight increase in the last time points in the *ppa2*Δ mutant (Figures 2A,B). Hence, we conclude that, while both PP2A and Clp1 counteract mitotic phosphorylation, PP2A activity is the major contributor to CDK substrate dephosphorylation observed during a sustained mitotic arrest.

We next looked at cytokinesis induction in our mutants. Neither deletion of *clp1* or *ppa2* prevented septation in our experimental set up. Similar to the slight advancement in protein phosphorylation, the cytokinesis phenotypes (*cut* and unequal segregation) were more obvious in the *clp1*Δ strain after 2 h of arrest (Figures 2C,E).

Remarkably, in the *ppa2*Δ mutant, septa could already be observed as early as after 1 h of *slp1* repression, and this phenotype was exacerbated over time with many cells exhibiting multiple septa (Figures 2D,F). Indeed, these cells were clearly impaired in preventing cytokinesis during the arrest, as judged by the low percentage of cells arrested in metaphase that did not contain a septum *vs*. those containing one or more septa. This effect was not solely due to an advancement in the G2/M transition in the *ppa2*Δ mutant, since *clp1*Δ and *ppa2*Δ cells had a similar number of metaphase-arrested cells 1 h into the arrest (approximately 60%) but very few *clp1*Δ cells had undergone *cut*/unequal segregation in comparison (compare the 1 h timepoint in Figures 2C,D).

Importantly, the sustained phosphorylation of CDK substrates (which presumably include targets of CDK involved in cytokinesis) was not sufficient to prevent septation in the *ppa2*Δ mutant. Therefore, these results reinforce the idea that cytokinesis can take place in the presence of high CDK-dependent phosphorylation and suggest that additional mechanisms regulated by PP2A activity are required to block cytokinesis during a mitotic arrest.

### B55^Pab1^ and B56^Par1^ PP2A-Regulatory Subunits Have Opposite Effects in the Induction of Septation in Metaphase Arrested Cells

**FIGURE 3:**
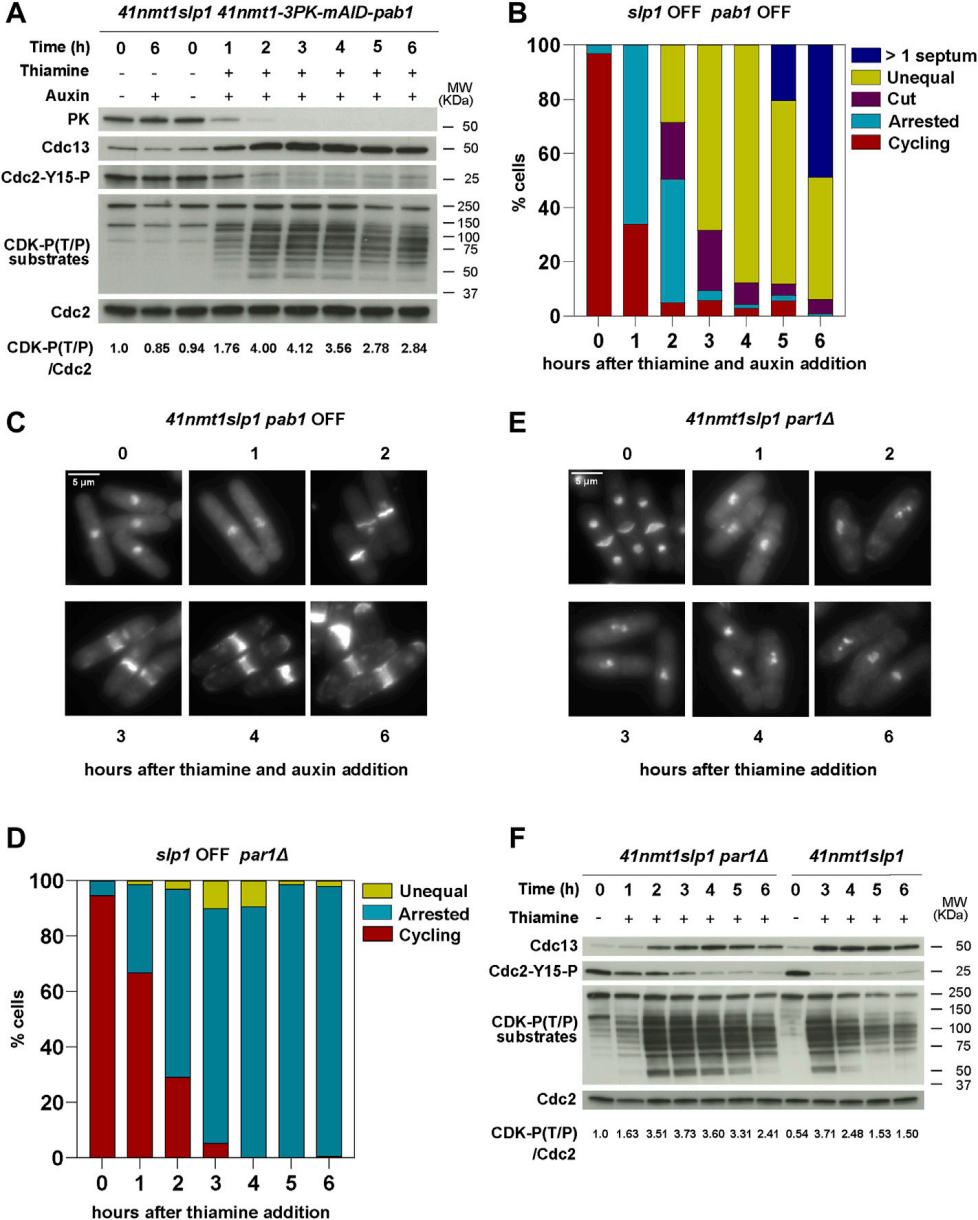
B55^Pab1^ and B56^Par1^ PP2A-regulatory subunits have opposite effects in the induction of septation in metaphase arrested cells. **(A–C)** The indicated strains were grown in EMM and then shifted to EMM with 0.5 mM 1-Naphthaleneacetic acid (auxin) for Pab1 degradation and/or 15 µm thiamine (*41nmt* promoter repressed) at 30°C. Samples were collected at the indicated time points for microscopy and western blot. **(A)** Depletion of Pab1 was detected by western blot against its N-terminal 3PK tag. See also Supplementary Figure S1. Cdc13 levels, phosphorylation at T/P sites in CDK substrates and Cdc2 phosphorylated at Y15 served as indicators of CDK activity and were detected by western blot. Cdc2 (PSTAIR) was used as a protein loading control. The numbers under the western blot indicate CDK-substrate Phosphorylation (P-T/P) quantification relative to Cdc2. **(B)** The percentage of cycling cells, metaphase-arrested cells, and arrested cells that underwent cytokinesis (cut or unequal) were determined at the indicated time points after the addition of thiamine to the culture. At least 200 cells were counted per time point. **(C)** Representative images of mitotic features distributions from DAPI and Blankophor-stained ethanol-fixed cells of *p41-nmt1:slp1p41-nmt1-3PK-miniAID-pab1* in **(B)**. Scale bar 5 µm. **(D–F)** *p41*-*nmt1:slp1 par1Δ* cells were grown exponentially in EMM (*p41-nmt1* promoter induced) and shifted to EMM +T (*p41-nmt1* promoter repressed) at 30°C. Samples were collected at the indicated time points for microscopy and western blot. **(D)** The percentage of cycling cells, metaphase-arrested cells, and arrested cells that underwent cytokinesis (cut or unequal) were determined at the indicated time points after the addition of thiamine to the culture. At least 200 cells were counted per time point. **(E)** Representative images of mitotic features distributions from DAPI and Blankophor-stained ethanol-fixed cells of *p41-nmt1:slp1 par1Δ* in **(D)**. Scale bar 5 µm. **(F)** Cdc13 levels, phosphorylation at T/P sites in CDK substrates and Cdc2 phosphorylated at Y15 in *p41-nmt1:slp1* and *p41-nmt1:slp1 par1Δ* strains were detected by western blot. Cdc2 (PSTAIR) was used as a protein loading control. The numbers under the western blot indicate CDK-substrate Phosphorylation (P-T/P) quantification relative to Cdc2.

Deletion of *ppa2* affects both B55^Pab1^- or B56^Par1^-containing PP2A complexes. Therefore, we decided to investigate next the consequences of specifically depleting individual regulatory subunits. In the case of B56 (*par1*), we used the deletion mutant. However, for B55 (*pab1*), we used an AID (auxin-inducible degron)-tagged allele under the control of the *p41-nmt1* promoter, since the deletion mutant has a slower growth rate and morphology defects (Martín et al., 2017). In this background, *pab1* expression was terminated at the same time as that of *slp1* upon thiamine addition. Moreover, degradation of B55^Pab1^ was triggered upon treatment with the plant hormone Auxin (NAA, 1-Naphthaleneacetic acid) (Figures 3A and Supplementary Figure S1A). Only the combination of Auxin and Thiamine resulted in the complete depletion of Pab1 and its associated phenotypes (Figure 3A and Supplementary Figure S1B).

Repression of *slp1* in the conditional *pab1* mutant resulted in 60% of the cells arrested in metaphase after 1 h of thiamine addition (Figure 3B). As in the wild type background, this arrest was accompanied by Cdc13 accumulation and Cdc2-Tyr15 phosphorylation disappearance (Figure 3A). However, after 2 h, 50% of the cells depicted a *cut* phenotype or had undergone unequal segregation. After 3 h, 90% presented septation-associated phenotypes. By the end of the time course, all cells had undergone septation and in 50% of the cases presented multiple septa, resembling the phenotypes observed in the *ppa2*Δ mutant (Figures 3B,C). Although to a lesser extent compared to the *ppa2* deletion, depletion of B55^Pab1^ also resulted in the sustained phosphorylation of CDK substrates, which suggests that PP2A-B55^Pab1^ is partly responsible for the drop in phosphorylation observed in the wild type background (Figure 3A).

The deletion of *par1* is normally associated to a defect in cell separation and, as a result, cycling *par1*Δ cells have a high septation index (Kinoshita et al., 1993; Jiang and Hallberg, 2000; Stonyte et al., 2020). Nevertheless, depletion of *slp1* in the *par1*Δ mutant allowed for cells to arrest in metaphase and finalize cell separation (see the disappearance of a 4C DNA peak in Supplementary Figure S1C). In contrast to the *ppa2*Δ or the conditional *pab1* mutant, the metaphase arrest in the *par1*Δ mutant was slightly slower, but similar to the arrest observed in the early time points in the wild type background (compare Figures 3D,E with Figures 1E,D). Strikingly, the deletion of *par1* abrogated septation almost completely and cells remained with condensed chromosomes and virtually no septa for the duration of the time course (Figures 3D,E). CDK substrate phosphorylation was also altered by the absence of *par1*. These cells reached a maximal phosphorylation after 3 h of *slp1* repression, and this was sustained for longer, only decaying slightly in the last timepoint (Figure 3F). As in the wild type strain, Cdc13 accumulated and stayed constant in the *par1*Δ mutant. However, Cdc2-Tyr15 phosphorylation was more prominent in the mutant during the early time points (Figure 3F). Of note, the highest degree of phosphorylation in the *par1*Δ background was somehow lower than that attained in the wild type background, which suggests that *par1*Δ cells might enter mitosis with reduced CDK activity.

From these results we conclude that PP2A-B55^Pab1^ is required to prevent untimely cytokinesis during a prolonged arrest in metaphase. In contrast, PP2A-B56^Par1^ facilitates septation during the arrest and, in its absence, cytokinesis is blocked. In this regard, the phenotype of the *ppa2*Δ mutant is likely to reflect the loss of PP2A-B55^Pab1^.

Nevertheless, albeit their opposite effects in the induction of cytokinesis, both PP2A-B55^Pab1^ and PP2A-B56^Par1^ contribute to the steady decay in CDK-substrate phosphorylation that occurs upon a prolonged arrest in metaphase.

### Septation Is Induced in Metaphase-Arrested *par1*Δ Cells if CDK Activity Is Enhanced

**FIGURE 4:**
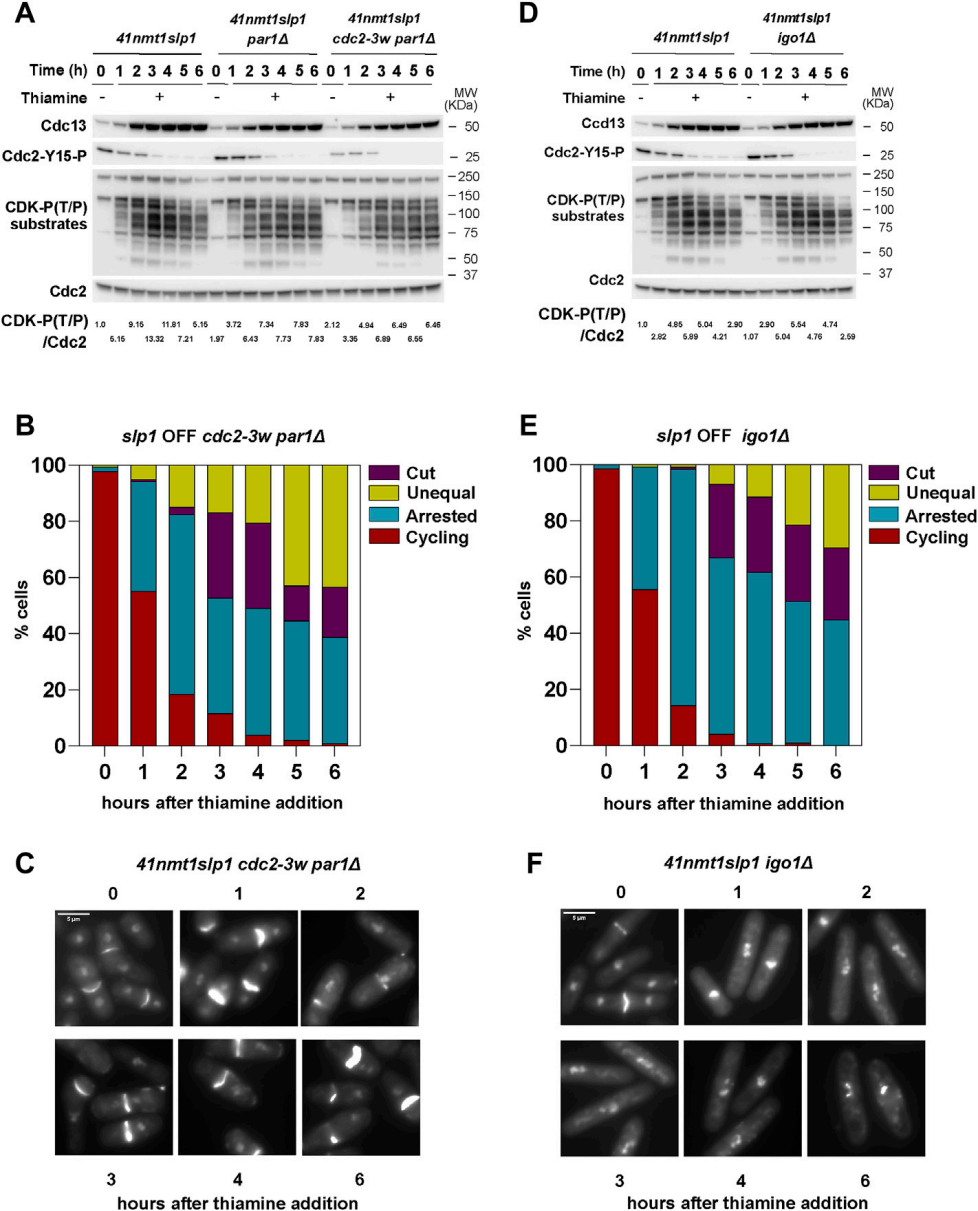
Interplay between CDK and PP2A-B55^Pab1^ in the regulation of septation. **(A–F)** Cells were grown exponentially in EMM (*p41-nmt1* promoter induced) and shifted to EMM +T (*p41-nmt1* promoter repressed) at 30°C. Samples were collected at the indicated time points for microscopy and western blot. **(A,B)** Cdc13 levels, phosphorylation at T/P sites in CDK substrates and Cdc2 phosphorylated at Y15 in *p41-nmt1:slp1*, *p41-nmt1:slp1 par1Δ and p41-nmt1:slp1 cdc2-3w par1Δ* **(A)** or *p41-nmt1:slp1* and *p41-nmt1:slp1 igo1Δ* strains **(B)** were detected by western blot. Cdc2 (PSTAIR) was used as a protein loading control. The numbers under the western blot indicate CDK-substrate Phosphorylation (P-T/P) quantification relative to Cdc2. **(C,D)** The percentage of cycling cells, metaphase-arrested cells, and arrested cells that underwent cytokinesis (cut or unequal) in *p41-nmt1:slp1 cdc2-3w par1Δ* **(C)** and *p41-nmt1:slp1 igo1Δ* **(D)** were determined at the indicated time points after the addition of thiamine to the culture. At least 200 cells were counted per time point. **(E,F)** Representative images of mitotic features distributions from DAPI and Blankophor-stained ethanol-fixed cells in **(C,D)**. Scale bar 5 µm.

PP2A-B55^Pab1^ prevents mitotic entry by counteracting CDK-dependent phosphorylation of Cdc25 and Wee1, thus disrupting the feedback loops that promote the abrupt activation of Cdc2:Cdc13 at the G2/M transition. Accordingly, *ppa2*Δ cells are shorter due to their premature entry into mitosis. *Clp1*Δ cells are also characterized by a semi-*wee* phenotype, indicative of a short G2 phase in this mutant. In contrast, *par1*Δ cells are slightly longer than wild type cells (Stonyte et al., 2020), and, in our experimental set up, they retained Cdc2-Tyr15 phosphorylation for longer (Figure 3F). Still, after 3 h of *slp1* transcriptional repression almost all *par1*Δ cells were arrested in metaphase but, compared to the wild type strain, very few had undergone untimely cytokinesis. Therefore, we reasoned that a different threshold of CDK activity might be required to induce early mitotic events (such as chromosome condensation) but that a higher threshold needs to be exceeded to induce cytokinesis during the arrest. In order to test this hypothesis, we generated a *p41-nmt1:slp1 par1*Δ strain containing the *cdc2-3w* allele (Fantes, 1981; Enoch et al., 1992), which makes Cdc2 activation independent of Cdc25. Phosphorylation of Cdc2-Tyr15 in this mutant was already lower when *slp1* was expressed (−Thiamine condition, Figure 4A), and it rapidly declined upon *slp1* repression. Similar to the wild type and *par1*Δ strains, Cdc13 accumulated and remained constant throughout the experiment. However, the maximal level of Cdc13 was reduced compared to the wild type strain, suggesting that in these cells the amount of cyclin required for mitotic-commitment is lower. In accordance, the level of CDK substrate phosphorylation was slightly reduced and, similar to the single *par1*Δ mutant, sustained until the end of the time course (Figure 4A). Strikingly, in the *cdc2-3w* background, *par1* deletion did not suppress cytokinesis and up to 60% of cells depicted *cut/*unequal segregation phenotypes after 6 h of *slp1* depletion (Figures 4B,C). Therefore, these results suggest that the inability of *par1*Δ cells to induce cytokinesis upon a prolonged mitotic arrest is in part the consequence of the inefficient activation of Cdc2:Cdc13 during mitotic entry.

### Deletion of *igo1* Delays Cytokinesis

The conserved Greatwall-ENSA pathway (Ppk18-Igo1 in fission yeast) inhibits PP2A-B55^Pab1^ at the G2/M transition through a mechanism of unfair substrate competition (Williams et al., 2014); the kinase Ppk18 phosphorylates Igo1, converting it in a PP2A-B55^Pab1^ substrate. However, its rate of dephosphorylation is so slow that it blocks the access of other PP2A-B55^Pab1^ targets.

The TORC1 pathway prevents Ppk18 activity and through this regulation mitotic entry is coupled to cell growth and the acquisition of an adequate cell size for division (Chica et al., 2016). Consequently, *igo1*-deleted cells are unable to adjust mitotic commitment in response to nutritional changes in the environment.

In *Xenopus laevis* and mammalian systems CDK1 activates Greatwall, thus bringing about the inhibition of PP2A-B55 as cells enter mitosis (Gharbi-Ayachi et al., 2010; Mochida et al., 2010; Rata et al., 2018). Since PP2A-B55 also blocks CDK1 activation, both complexes are thus mutually inhibitory. The same regulation has not been proven in fission yeast, but it is also thought to participate in the regulation of mitosis (Chica et al., 2016).

Our previous experiments indicated that, on the one hand, a high level of CDK activity favors untimely cytokinesis in metaphase-arrested cells. On the other hand, PP2A-B55^Pab1^ prevents septation and, in consequence, depletion of B55^Pab1^ results in the exacerbation of the cytokinesis phenotypes during a metaphase arrest (Figures 3B,C). Therefore, we reasoned that the CDK mediated inhibition of PP2A-B55^Pab1^ could account for the untimely induction of cytokinesis in our system. An expected corollary to this idea would be that *igo1* deletion would ameliorate the septation phenotypes in metaphase arrested cells. Indeed, loss of *igo1* resulted in a consistent decrease in the proportion of cells that had undergone cytokinesis during the arrest in metaphase, and this phenotype was more accentuated in the last time points (Figure 4E). It is worth noting that since the experiment was done in minimal media containing a rich source of nitrogen (EMM), we did not detect changes in cell size (Figure 4F) or the pattern of Cdc13 accumulation in the *igo1*Δ strain compared to the control (Figure 4D). Similarly, Cdc2-Tyr15 phosphorylation and CDK-substrate phosphorylation was comparable to that observed in the wild type strain upon depletion of *slp1* (Figure 4D).

From these results we conclude that Igo1-mediated inhibition of PP2A-B55^Pab1^ contributes to the induction of septation during a prolonged metaphase arrest. However, we cannot exclude additional mechanisms, including different modes of PP2A-B55^Pab1^ inhibition, that could also participate in the observed phenotypes.

### *Ppa2*Δ Cells Depict Hyperphosphorylation of Byr4

Septation in fission yeast is under the control of the septation initiation (SIN) pathway. The bipartite GAP composed of Cdc16 and Byr4 prevents activation of the cascade by promoting the conversion of GTP to GDP by the small GTPase Spg1. During mitosis, the Polo-like kinase Plo1 and Cdc2:Cdc13 (Johnson and Gould, 2011; Rachfall et al., 2014) phosphorylate and inhibit Byr4, hence promoting activation of the top of the SIN. However, CDK activity prevents activation of downstream components of the pathway by impairing the recruitment of Cdc14:Sid1 to the spindle pole body (Guertin et al., 2000).

Given that deletion of *par1* completely abrogated septation during the arrest, while *ppa2* deletion exacerbated it, we next examined the pattern of phosphorylation of Byr4 upon *slp1* depletion in the different strain backgrounds.

**FIGURE 5:**
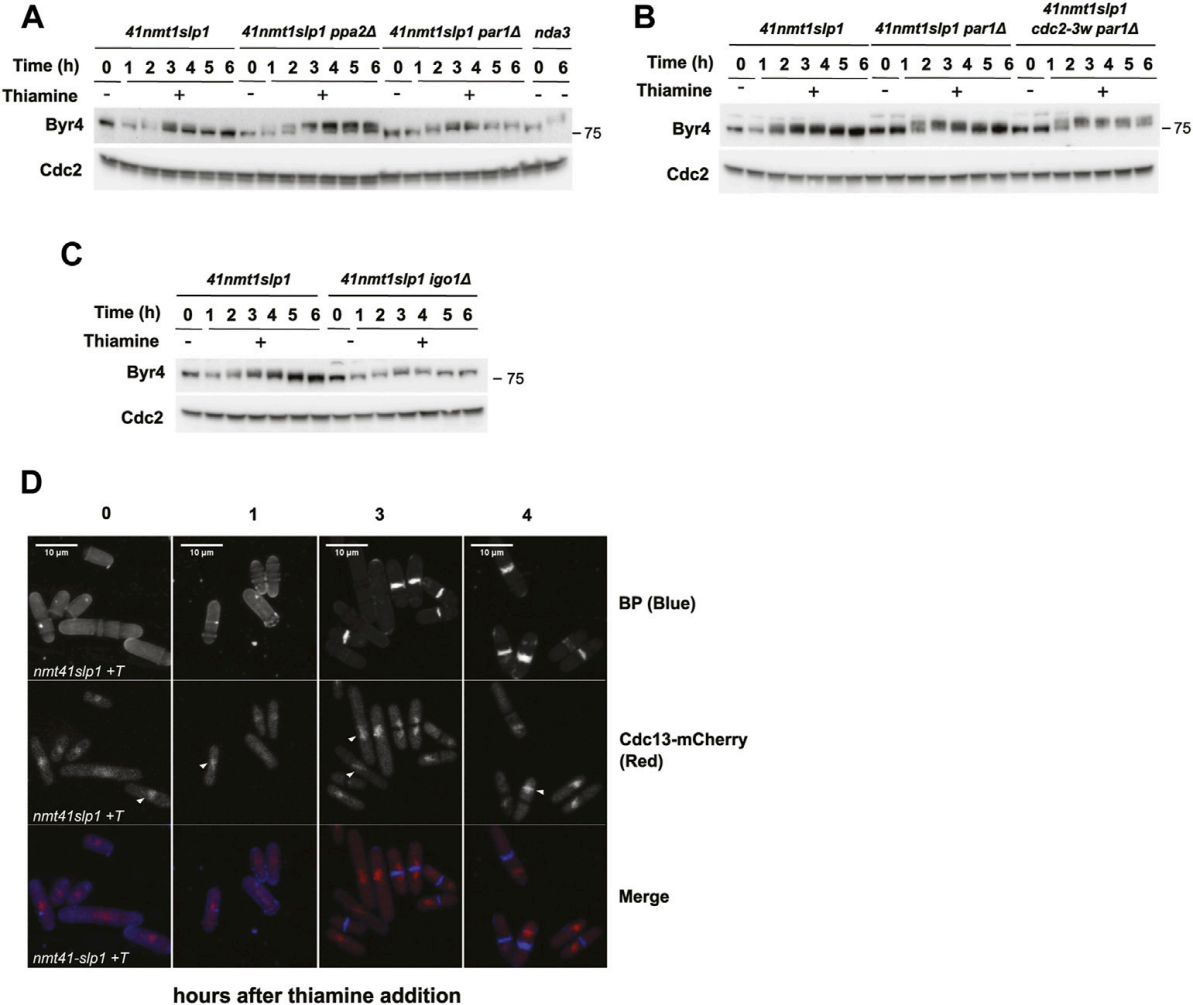
Dynamic Byr4 phosphorylation and Cdc13 localisation during a sustained metaphase arrest. **(A–D)** Cells were grown exponentially in EMM (*p41-nmt1* promoter induced) and shifted to EMM +T (*p41-nmt1* promoter repressed) at 30°C. Samples were collected at the indicated time points for microscopy and western blot. For the *nda3-3KM311* mutant, cells were grown at 30 and then shifted to 18°C for 6 h. **(A,C)** Byr4 levels and phosphorylation (band shift) were detected by western blot in *p41-nmt1:slp1*, *p41-nmt1:slp1 ppa2Δ*, *p41-nmt1:slp1 par1Δ and nda3-3KM311* mutant **(A)** *; p41-nmt1:slp1*, *p41-nmt1:slp1 par1Δ and p41-nmt1:slp1 cdc2-3w par1Δ* **(B)** *and p41-nmt1:slp1 and p41-nmt1:slp1 igo1Δ* **(B)** or *p41-nmt1:slp1* and *p41-nmt1:slp1 igo1Δ* strains **(C)** in EMM -T and EMM +T at the indicated time points. Cdc2 (PSTAIR) was used as a protein loading control. **(D)** Representative images of *cdc13mCherry* localization and Blankophor-stained ethanol-fixed cells upon *slp1* repression by thiamine. Scale bar 5 µm.

In the wild type background (*p41-nmt1-slp1*), Byr4 phosphorylation (as judged by the appearance of a slower migrating band in the western blot) increased as cells became arrested in metaphase. Maximal phosphorylation was achieved after 2 h of *slp1* repression, coincident with the early cytokinetic events. The level of phosphorylation was comparable to that achieved during a metaphase arrest in a *nda3-KM311* mutant. Surprisingly, Byr4 phosphorylation was not sustained and, soon after the peak, started decreasing, not being detectable by 5–6 h of metaphase arrest (Figure 5A). In contrast, in the *par1*Δ as in the *par1*Δ *cdc2-3w* background, Byr4 phosphorylation remained for longer (Figure 5B). In fact, in the *par1*Δ *cdc2-3w p41-nmt1:slp1* mutant the phosphorylation shift upon *slp1* depletion was more marked than in the *p41-nmt1:slp1* or *par1*Δ *p41-nmt1:slp1* strains and it was still evident 6 h into the arrest (Figure 5B). Finally, silencing of *slp1* in the *ppa2*Δ *p41-nmt1:slp1* mutant resulted in the highest degree of Byr4 phosphorylation and this was sustained throughout the arrest (Figure 5A). In comparison, the *igo1*Δ *p41-nmt1:slp1* strain behaved similarly to the *p41-nmt1:slp1* strain with regard to the pattern of Byr4 phosphorylation during the arrest (Figure 5C).

PP2A-B55^Pab1^ has been suggested to counteract Plo1 and CDK-dependent phosphorylation of Byr4 (Lahoz et al., 2010), thus repressing the SIN. Indeed, our results point in that direction since removal of *ppa2* (but not *par1*) led to a substantial increase in the level of Byr4 phosphorylation. Moreover, the resemblance between the *ppa2*Δ and *par1*Δ *cdc2-3w* strains reinforces the idea that enhanced CDK activity in the latter mutant results in suppression of PP2A-B55^Pab1^ activity and induction of septation.

### Cdc13 Localization During a Prolonged Mitotic Arrest

As mentioned above, Byr4 phosphorylation decreased in the wild type strain as cells initiated septation. This finding was surprising, since we expected phosphorylation to remain high in metaphase-arrested cells with sustained CDK activity.

A possible explanation for this could be that, upon a prolonged arrest, Cdc13 delocalizes from the SPB and can no longer promote the phosphorylation of Byr4. A recent work from the Nurse laboratory has shown that cells carrying a mutated allele of Cdc13 which impairs its SPB localization depict defective phosphorylation of a number of mitotic substrates (Basu et al., 2020). Among the substrates identified in this study they found Sid2, the last kinase in the SIN cascade.

Byr4 phosphorylation activates the SIN pathway, and therefore one would assume that it should remain phosphorylated for septation to occur. However, a plausible hypothesis would be that Byr4 only needs to be transiently inhibited to allow for the early activation of the SIN, and that subsequent displacement of Cdc13:Cdc2 from the SPB would facilitate recruitment of Sid1:Cdc14 and firing of the pathway.

Hence, we next investigated Cdc13 localization during the arrest, using an mCherry tagged allele. Cdc13-mCherry signal accumulated in cells arrested in metaphase and, in agreement with our western blot analysis, it reached a plateau after 3 h of thiamine addition (Figure 5D). The localization was mainly nuclear and we could also detect specific signal in a region compatible with the mitotic spindle (arrowheads in Figure 5D). Nevertheless, we did not observe obvious changes in the pattern of localization in cells arrested in metaphase compared to those that had undergone cytokinesis. Therefore, we conclude that Cdc13 displacement is not the underlying cause for untimely cytokinesis in metaphase-arrested cells.

The fact that Byr4 phosphorylation is not sustained during the arrest is however interesting, and indicates that additional mechanisms contribute to its tight spatial and temporal regulation.

### Proteins Involved in Cytokinesis Accumulate in Metaphase Arrested Cells

We next decided to investigate changes in the proteome composition of metaphase-arrested cells that could explain the induction of cytokinesis upon extended periods of *slp1* depletion.

**FIGURE 6:**
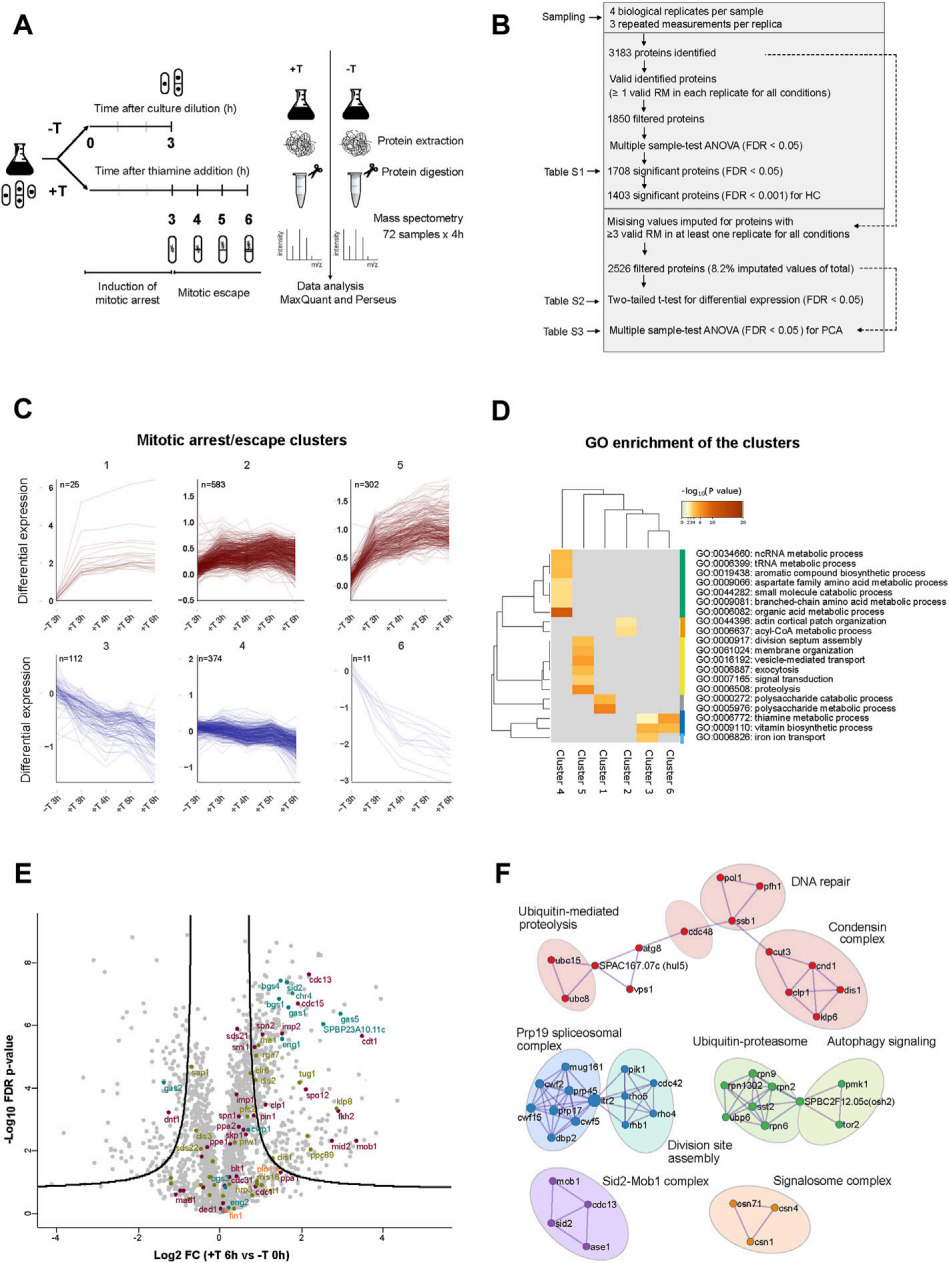
Proteins involved in cytokinesis accumulate in metaphase arrested cells. **(A)** Schematic of the MS based label-free proteomics workflow to quantify the relative protein levels between samples of *p41-nmt1:slp1* cells non-treated (−T) and treated with thiamine (+T) in a range of 3–6 h, to characterise the proteome of the mitotic arrest and escape induced by the thiamine-regulatable *slp1* expression system. The workflow is divided in four parts: (i) sampling (ii) protein extraction and digestion (iii) MS analysis (iv) identification and quantification of proteins using MaxQuant and data interpretation using Perseus. **(B)** The numbers of protein analysis at each stage of the analysis applied to the experimental dataset composed by four biological replicates and three repeated measurements (RM) for each. The initial dataset contains all proteins with at least 1 valid RM in each replicate for all conditions (Supplementary Table S1). The resultant significant proteins from Multiple sample-test ANOVA (FDR < 0.0001) were used for the hierarchical clustering analysis (HC). The second dataset contains all proteins with ≥3 valid measurements in at least one replicate for all conditions as basis for the differential expression analysis (Supplementary Table S2) and PCA (Supplementary Table S3). **(C)** Mitotic/arrest clusters of proteins extracted from HC. 6 clusters in total. Upper clusters contain proteins with a positive dynamic expression and lower clusters contain proteins with negative dynamic expression over the time course of *slp1* repression (Supplementary Table S1). **(D)** Top GO terms enriched in cluster-wise comparisons. Metascape; GO BP annotations, background gene list (Supplementary Table S1), and a hypergeometric test with Benjamini–Hochberg correction. All GO terms are listed in Supplementary Table S4. **(E)** Volcano plot. The log2 FC indicates the mean expression level for each protein. Each dot represents one protein. The line represents a cut-off of FDR < 0.01 with a S0 = 0.5. Beyond the line, black dots represent significant hits between EMM T0 and EMM +T 6 h. Dots are coloured based on GO annotations: purple, cell cycle; green, chromosome segregation; orange, cytokinesis and septation and blue, 1-3 ß-glucanase activity. **(F)** PPI network of the proteins upregulated after thiamine addition (6 h). Circles indicate nine hubs of protein-protein interactions. Annotations are based on MCODE PPI and curated GO annotations in Pombase.

For that, we carried out a label-free proteomic analysis of *p41-nmt1:slp1* cells, in the absence or presence of thiamine, over a time course (Figure 6A). We chose to take samples at time 0 h and 3 h, 4 h, 5 h and 6 h since those where the time points when the induction of septation became more prominent. Principal component analysis (PCA) of the samples showed a high degree of reproducibility between biological replicates with a greater difference between non-treated and treated samples (Supplementary Figure S2A). Upon digestion and mass spectrometry analysis (see *Methods* for details) we identified 3183 proteins. A first filter was applied to select proteins for which at least one valid measurement in each replicate, in each condition had been identified (Figure 6B). This filter yielded 1,850 hits that were subsequently subject to ANOVA multi-sample test. This reduced the number of hits to 1,708 proteins that were statistically significant under a 5% FDR cut-off (Figure 6B and Supplementary Table S1). Hierarchical clustering of the top hits (1,403 proteins ≤0.1% FDR) in which each sample was normalized to the control sample (0 h), revealed six different dynamical clusters (Supplementary Figure S2B and Figure 6C). Proteins in clusters 1 and 2 were enriched in cells arrested in metaphase (+thiamine) vs. non-arrested cells (3 h–thiamine), but did not increase further as the arrest persisted (cluster 1) or even decreased in the last time point (cluster 2). In contrast, proteins in cluster 5 did show a steady increase throughout the metaphase arrest. Proteins in clusters 3, 4 and 6 showed the opposite trend, having a higher level of expression in non-arrested vs. arrested cells. Gene ontology analysis of the different clusters indicated an enrichment of the terms *septation, cell signalling, vesicle-mediated transport and proteolysis* for cluster 5 (Figure 6D). Finally, a protein-protein interaction (PPI) network analysis of the top hits revealed substantial enrichment of several functional groups involved in cell cycle regulation, chromatin remodelling and RNA metabolism (Supplementary Figure S2C).

In parallel, we applied a second, more relaxed filter to the proteomic data, in order to unveil hits that were not considered in the first analysis due to its higher astringency. In this case, we imputed missing values for proteins with at least three valid measurements in at least one replicate in each condition. This treatment only entailed the imputation of 4,978 values out of a total of 60,624 values (8.2%) in a total of 2,526 proteins. For each timepoint in the time course, we performed a two-tailed *t*-test and considered statistically significant proteins with a two-fold change and FDR 1% cut-off compared to the 0 h control (Figure 6B and Supplementary Table S2). Within the group of upregulated proteins, we detected an overrepresentation of proteins involved in septation and cytokinesis during the time course of slp1 repression (Supplementary Figures S3B,D,E), as well as in cell cycle, chromosome segregation and 1-3 β-glucanase activity (Figure 6E and Supplementary Figures S3A,C). In addition, when we explored the PPI network of the upregulated proteins, we obtained clusters of interacting proteins involved in *division site assembly, Sid2-Mob1 complex, condensin complex, Ubiquitin-Proteasome* and *ubiquitin-mediated proteolysis* among others (Figure 6F). Notably, these groups show strong correspondence with the enriched terms associated with the dynamics of cluster 5 (Figure 6D).

Upregulation of these proteins could be the consequence of their altered degradation upon depletion of the APC/C activator Slp1. Hence, we searched the list of upregulated proteins for known degradation signals recognised by Slp1, that is, destruction box (DB, RxxLxxxxN) and KEN box (KENxxxN). Out of the 285 proteins significantly upregulated, only 26 contained a DB (either putative or experimentally confirmed), 2 contained a KEN box and 1 contained both (Supplementary Figure S3F).

This analysis indicates that the metaphase arrest brings about substantial changes in the proteome of fission yeast cells, with a specific enrichment for proteins involved in cytokinesis and septation, in addition to cell cycle regulators. Moreover, the accumulation of these proteins cannot solely be alleged to a block in their degradation upon depletion of Slp1, since many of them lack a recognisable degron. Finally, these results suggest that the upregulation of cytokinesis-related proteins could pave the way for septation after a prolonged arrest in metaphase.

### The Mitotic Transcriptional Wave Influences the Behavior of Cells Arrested in Metaphase

Our proteomic analysis highlighted the induction of a number of cytokinesis regulators in cells that had been arrested in metaphase for long periods of time. In fission yeast, as in other organisms, cell cycle-associated transcriptional waves accompany and are required for adequate cell cycle progression (reviewed in McInerny, 2011). The PBF (pombe cell cycle box-binding factor), a complex containing the Forkhead transcription factors Fkh2 and Sep1 and the MADS box protein Mbx1 binds the promoter of genes expressed during mitosis. Fkh2 is thought to act as a general repressor, whereas Sep1 activates transcription of its target genes. In addition, the RFX transcription factor Sak1 was shown to positively regulate the expression of the same mitotic cluster (Garg et al., 2015). One of the targets of these transcriptional complexes is the transcription factor Ace2, which in turn regulates the expression of genes required for primary septum degradation and cell separation. Interestingly, among the proteins upregulated in our experimental set up we could detect known targets of these transcription factors, including Sid2, Spo12, Plo1, Cdc15, Ace2, Eng1, Gas1 and Gas5 (Figure 6E and Supplementary Figures S3A,C).

**FIGURE 7:**
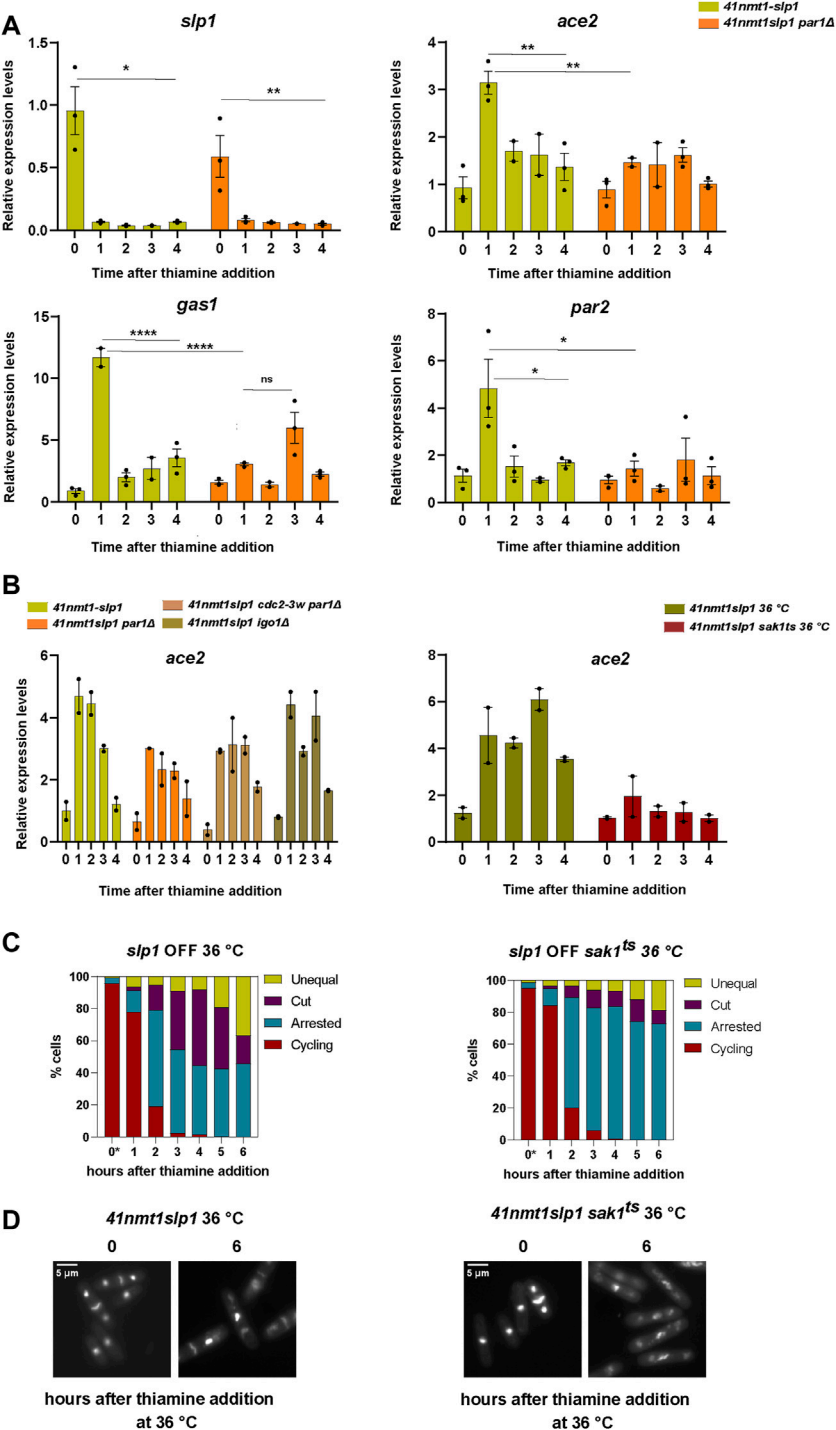
The mitotic transcriptional wave influences the behaviour of cells arrested in metaphase. **(A–D)** Cells were grown exponentially in EMM at 30°C or 25°C (*p41-nmt1* promoter induced) and shifted to EMM +T (*p41-nmt1* promoter repressed) at 30°C or 36°C. Samples were collected at the indicated time points for mRNA expression and microscopy. **(A)** mRNA expression of *slp1, ace2, gas1 and par2* in wild type and in *p41-nmt1:slp1 or p41-nmt1:slp1 par1Δ* cells. Expression is relative to actin and was determined by qPCR. Mean and SEM of three biological replicate are shown. Statistical significance was determined by a two-way ANOVA with Tukey’s multiple comparisons test. ns: *p* > 0.05; *: *p* ≤ 0.05; ** ≤ 0.01; ****: *p* ≤ 0.0001. **(B)** Left panel. mRNA expression of *ace2* in *p41-nmt1:slp1 or p41-nmt1:slp1 par1Δ*, *p41-nmt1:slp1 cdc2-3w par1Δ and p41-nmt1:slp1 igo1Δ* cells. Expression is relative to actin and was determined by qPCR. Mean and SEM of at least two biological replicates are shown. Right panel. mRNA expression of *ace2* in *p41-nmt1:slp1 or p41-nmt1:slp1 sak1ts* cells. Expression is relative to actin and was determined by qPCR. Mean and SEM of at least two biological replicates are shown. **(C)** The percentage of cycling cells, metaphase-arrested cells, and arrested cells that underwent cytokinesis (cut or unequal) in *p41-nmt1:slp1 (left panel)* and *p41-nmt1:slp1 sak1ts* (right panel) at each indicated time point after thiamine addition. (*) cells grown at 25°C. At least 200 cells were counted per time point. **(D)** Representative images of mitotic features distributions from DAPI and Blankophor-stained ethanol-fixed cells in **(C)**. Scale bar 5 µm.

In order to test if transcriptional upregulation could lie behind the accumulation of these proteins, we analyzed their expression pattern during the course of our experiment by real time PCR. Indeed, we could observe a sharp increase in the expression of *gas1*, *ace2* and *par2* as cells became arrested in metaphase upon depletion of *slp1* (Figure 7A). Interestingly, expression of the same genes was significantly reduced in the *p41-nmt1:slp1 par1*Δ mutant.

Hyperactivation of Cdc2 by means of the *cdc2-3w* allele resulted in the partial induction of septation in metaphase arrested *p41-nmt1:slp1 par1*Δ (Figures 4B,C). Hence, we next tested whether the same allele could rescue the expression of mitotic transcripts in the *par1*Δ background. Expression of this cluster of genes depends on the achievement of a certain threshold of CDK activity (Banyai et al., 2016). However, we did not observe a significant change in the expression of *ace2* (hereafter our readout for mitotic transcription) upon hyperactivation of *cdc2* (Figure 7B, left panel)*.*

Favoring the activity of PP2A-B55^Pab1^ through the deletion of *igo1,* which partially rescued the induction of cytokinesis during the metaphase arrest, did not result in major changes in the transcription of *ace2* (Figure 7B, left panel).

It might be possible that CDK activity in the *cdc2-3w par1*Δ allele is not sufficient to fully activate the mitotic transcriptional program. However, the observation that *igo1* deletion does not have a great impact in the expression of (at least) *ace2*, while being sufficient to hinder septation to a greater extent than the combination of the *cdc2-3w* and *par1*Δ alleles (compare Figures 4B,E), argues against this possibility. Therefore, we believe that PP2A-B56^Par1^ is required for the activation of the mitotic transcriptional wave and that this role is independent of the regulation of CDK activity.

Finally, as a proof of principle, we investigated the phenotype of a *p41-nmt1:slp1 sak1* ^*ts*^ allele [*sak1-891* (Yuasa et al., 2004; Garg et al., 2015)]. As expected, inactivation of Sak1 at the restrictive temperature strongly reduced the expression of *ace2* (Figure 7B, right panel). This impairment in the expression of mitotic genes was accompanied by a higher proportion of cells that remained arrested in metaphase without inducing cytokinesis (Figures 7C,D).

All in all, we conclude that mitotic transcription contributes to the induction of cytokinesis in metaphase-arrested cells upon depletion of *slp1.* In addition, we show that PP2A-B56^Par1^ is required for the activation of this cluster and that this function is partially responsible for the suppression of septation in the *p41-nmt1:slp1 par1*Δ mutant.

## Discussion

During a normal cell cycle, mitosis and cytokinesis need to be coupled, so that a cell does not attempt to divide unless its chromosomes have been segregated and pulled towards opposite ends of the cell by the mitotic spindle. In fission yeast, this coordination is achieved because the APC/C bound to its activator Slp1 (Cdc20) is responsible for the degradation of the mitotic Cyclin B (Cdc13) (Yamano et al., 1996) and Securin (Cut2), the inhibitor of Separase (Funabiki et al., 1997). The mitotic CDK complex Cdc13:Cdc2 prevents cytokinesis by repressing the activity of the SIN (Septation Initiation Network) cascade (Guertin et al., 2000). Therefore, during metaphase, before APC/C^Slp1^ activation, CDK activity is high and prompts early mitotic events associated to the phosphorylation of mitotic substrates. Chromosome segregation cannot take place while APC/C^Slp1^ is not active, since Securin-bound Separase cannot cleave the cohesin complexes that hold sister chromatids together. The delay in the next cell cycle transition, metaphase to anaphase, is imposed by the mitotic checkpoint (*AKA* spindle assembly checkpoint or SAC), which prevents activation of the APC/C^Slp1^ until all kinetochores are amphitelically attached to the spindle.

Once this condition has been fulfilled, APC/C^Slp1^-mediated degradation of Cdc13 and Securin brings about chromosome segregation, mitotic dephosphorylation and cytokinesis. This regulation, which is conserved through evolution, has been exploited for the study of mitotic progression and, in human cells, for the treatment of cancer. In mammalian cells, suppression of APC/C^Cdc20^ activity following activation of the mitotic checkpoint or after chemical inhibition with APC/C-targeting drugs (e.g., Apcin and ProTAME) arrests cells in metaphase. A majority of cells whose mitotic progression is hindered eventually undergo apoptosis (Sloss et al., 2016). However, an alternative fate is mitotic slippage, a process by which cells skip cytokinesis and exit mitosis without an intervening round of chromosome segregation (Brito and Rieder, 2006). This strategy, while not optimal, prevents apoptosis and provides the single cell with an opportunity for survival. From the organismal point of view, mitotic slippage can have deleterious effects since it increases the cell’s genomic instability, a trait that contributes to the evolution of tumors.

Here, we show that fission yeast cells that have been arrested in metaphase through the depletion of the APC/C activator Slp1 can only remain in this state for a certain period of time. In contrast to human cells, *S. pombe* cells do not skip cytokinesis before exiting mitosis and entering G1. Fission yeast cells do not attempt to segregate their chromosomes, which remain condensed throughout the arrest. However, mitotic phosphorylation progressively decays and cytokinesis is induced. This behavior had been noticed for many years in conditional mutants of the APC/C as well as for Separase (*cut1*) and Securin (*cut2*) (Uzawa et al., 1990; Samejima et al., 1993). These mutants undergo cytokinesis having failed to segregate their chromosomes, which results in their tearing by the ingressing septum (hence the name *cut, cell untimely torn*). Interestingly, in our hands, a considerable portion of the cells that undergo cytokinesis do not “cut” their chromosomes. Instead, the nucleus seems to be pushed to one side of the cell, giving rise to an anucleated compartment and a compartment retaining the undivided nucleus. Recently, work from the Toda laboratory has uncovered a mechanism that facilitates the drifting of the nucleus in order to prevent “cutting” of the DNA and mitotic catastrophe (Yukawa et al., 2021). This process, that is highly dependent on the actin cytoskeleton, reinforces the idea that evolution has developed alternative mechanisms to give cells a last chance of survival when the final step in the cell cycle, mitosis, cannot be completed. While human cells skip cytokinesis and become polyploid, fission yeast cells attempt to displace their nucleus and undergo cytokinesis, with more or less success.

How these mechanisms are triggered is an interesting question. On the one hand, understanding this regulation in human cells and how to prevent it can lead to new treatment strategies for cancer. On the other hand, from a systems biology perspective, it implies the presence of feed-forward mechanisms that bring about mitotic exit if the cell has been in mitosis for a long period of time. Given that mitotic events rely on the activity of the mitotic CDK complex, it is likely that such feed-forward mechanisms would also depend on this complex. That is the case for early mitotic exit events in budding yeast, where Cdc28 is responsible for the release and activation of its counteracting phosphatase, Cdc14 during the metaphase to anaphase transition (Azzam et al., 2004; Queralt et al., 2006). Importantly, mitotic slippage only occurs if mitotic progression has been prevented for a prolonged period, which would call for a delay in the engagement of the feed-forward mechanism. Such a delay could be imposed if a higher threshold of CDK activity was required to bring about mitotic slippage than to promote mitotic entry. This could be achieved through the stockpiling of mitotic cyclin and accumulation of active mitotic CDK complex as cells become arrested in metaphase. Alternatively, the delay could be the consequence of the expression of proteins involved in the next phases of the cell cycle and their sufficient accumulation in metaphase arrested cells.

Our experiments suggest that both possibilities contribute to the untimely induction of cytokinesis. Importantly, we demonstrate the involvement of phosphatase activity in the regulation of this process. Cells lacking the activity of PP2A-B56^Par1^ (*par1*Δ) enter mitosis and arrest with condensed chromosomes and enhanced mitotic phosphorylation upon depletion of *slp1*. However, CDK activity in these cells is somewhat compromised, as judged by the retention of Cdc2-Tyr15 phosphorylation in the early time points and a lower level of substrate phosphorylation compared to the control strain (Figure 4A). As a result, *par1*-deleted cells do not arrest as readily as the control strain. Nevertheless, their block is complete after 3 h of thiamine addition, indicating that sufficient CDK activity is achieved in this genetic background to enter mitosis and carry out early mitotic events. Concomitant mutation of *cdc2* to the hyperactive allele *cdc2-3w* partially rescued the *par1*Δ phenotype (compare Figures 3E, 4B). In this mutant, Tyr15 phosphorylation is markedly reduced, which lowers the threshold of Cdc13 required for mitotic entry.

The behavior of the single *par1*Δ mutant during the arrest is reminiscent of the stable intermediate prophase state reported by Rata et al. (2018) in mammalian cells. In their model, partial inhibition of CDK activity during the G2/M transition results in cells arrested in a prophase-like state, with a rounded cell morphology, partially-condensed chromosomes and intermediate levels of Tyr15 phosphorylation. Importantly, this intermediate state relies on the existence of two interlinked feedback systems involving CDK and PP2A-B55 activities. In this model, PP2A-B55 inhibits CDK through Wee1-mediated Tyr15 phosphorylation, whereas CDK inhibits PP2A-B55 through the activation of the Greatwall/ENSA pathway. As a consequence, the system is bistable and shows hysteresis, responding differently to CDK inhibition in interphase than in mitosis. Interestingly, in their model, if Wee1 activity is blocked, this intermediate state is no longer observed. In this regard, our double mutant *cdc2-3w par1*Δ, where CDK activity cannot be prevented through Tyr15 phosphorylation would result in a scenario similar to that of Wee1 inhibition.

How PP2A-B56^Par1^ impinges in CDK activation during the G2/M transition is still an outstanding question that we aim to address in the future. The *par1* deletion strain depicts a phenotype of slow growth and, although the average cell size of this mutant is only slightly larger compared to the wild type control, it shows a higher variability in cell size at division (Stonyte et al., 2020). One possible explanation that could link the cell size variability and increased Cdc2-Tyr15 phosphorylation in the *par1* mutant could be related to persistent presence of the NDR-kinase Pom1 in the middle of the cell. Pom1 participates in a pathway linking cell size and mitotic commitment. During G2 phase, a gradient of this kinase forms, accumulating at the cell tips and dissipating at the cell equator as the cell grows. Pom1 present at the cell middle prevents mitotic entry by repressing the Wee1 inhibitor Cdr2. Thus, not until its levels at this location drop below a certain threshold after the cell has reached an adequate size can it enter mitosis (Martin and Berthelot-Grosjean, 2009; Moseley et al., 2009). Nevertheless, we cannot exclude a direct involvement of PP2A-B56^Par1^ in the pathways regulating Tyr15 phosphorylation.

Another striking parallelism between our fission yeast model system and mammalian cells is the fact that PP2A-B55^Pab1^ inhibition seems to play a central role in the control of mitotic progression. In our experimental set up, deletion of either *ppa2* (the main catalytic subunit in PP2A complexes) or depletion of B55^Pab1^ led to the enhancement of the cytokinesis phenotypes. This observation indicates that PP2A-B55^Pab1^ plays an active role preventing cytokinesis and, in its absence, uncontrolled septation takes place. Taking this observation and the model of mutual inhibition between CDK and PP2A-B55 into account, we reasoned that CDK hyperactivation during the mitotic arrest ultimately leads to PP2A-B55^Pab1^ blockade, contributing to the induction of cytokinesis. In support of this idea, deletion of *igo1* (the fission yeast ENSA counterpart) which disrupts the PP2A-B55^Pab1^ negative feedback, could partially prevent the untimely septation phenotype upon *slp1* depletion*.*

Notably, although the deletion of *ppa2* results in the downregulation of both PP2A-B55^Pab1^ and PP2A-B56^Par1^ complexes, this mutant phenocopies the depletion of B55^Pab1^. This result indicates that PP2A-B55^Pab1^ loss is epistatic to *par1* deletion. Moreover, it reinforces the idea that the absence of cytokinesis in the *par1*Δ mutant is a reflection of its inability to engage the CDK-mediated PP2A-B55^Pab1^ negative feedback loop.

Both PP2A-B55^Pab1^ and PP2A-B56^Par1^ have been proposed as negative regulators of the Septation Initiation Network (SIN) (Jiang and Hallberg, 2000; Le Goff et al., 2001; Lahoz et al., 2010; Goyal and Simanis, 2012). However, in our experimental conditions, only the loss of PP2A-B55^Pab1^ activity brings about cytokinesis. The SIN cascade is under the control of CDK activity, which promotes activation of the upper part of the cascade (through inhibition of Byr4) while preventing the recruitment of downstream elements. PP2A-B55^Pab1^ was suggested as the Byr4 phosphatase, given that its inactivation could rescue the activity of the SIN GTPase Spg1 in the absence of Etd1 (Lahoz et al., 2010). It could be reasoned that loss of this mechanism of SIN repression upon depletion of B55^Pab1^ would not result in cytokinesis induction as long as CDK activity was blocking downstream events. However, it was also shown that PP2A-B55^Pab1^ prevents the activation of the small GTPase Rho1, a downstream target of the SIN whose overexpression rescues defective SIN signalling (Alcaide-Gavilán et al., 2014). Similarly, mutation of *pab1* could bypass the requirement for Sid2-Mob1 (the downstream kinases in the SIN) and promote septation in the absence of Etd1 and SIN signalling.

In addition to the induction of cytokinesis, the persistent arrest eventually led to the dephosphorylation of mitotic CDK-substrates. Given that Cdc13 was not degraded and the CDK complex was presumably active (considering that Tyr15 remained dephosphorylated), we decided to analyze the consequences of impaired phosphatase activity. We tested mutants of the Cdc14 homolog, *clp1,* as well as of the different subunits of PP2A: the regulatory subunits, *pab1* and *par1*, as well as the catalytic subunit *ppa2*. Loss of either phosphatase resulted in a more sustained phosphorylation of CDK substrates, suggesting cooperativity between phosphatases. Recently, a comprehensive phospho-proteomic study in budding yeast has shown that PP2A subcomplexes (PP2A-B55^Cdc55^ and PP2A-B56^Rts1^) together with Cdc14 are responsible for the dephosphorylation of substrates during mitotic exit (Touati et al., 2019). In our hands, deletion of *ppa2* resulted in the strongest effect, suggesting that in fission yeast as in budding yeast, PP2A subcomplexes are major contributors to mitotic dephosphorylation. At the moment we do not know whether high CDK activity is the trigger for the activation of these phosphatases or whether additional events secondary to the mitotic arrest facilitate the dephosphorylation of substrates observed in our experiments. Mitotic CDK activity indirectly promotes the transcription of a second B56 regulatory subunit, *par2*, which is part of the mitotic cluster (Suárez et al., 2015; Banyai et al., 2016). In this manner, CDK could be contributing to phosphatase activation. However, previous studies have shown that, during a normal mitosis, high CDK activity precludes the activities of PP1, PP2A-B55^Pab1^ and PP2A-B56^Par1^ (Grallert et al., 2015) as well as of Clp1 (Wolfe et al., 2006) until the metaphase to anaphase transition, when Cdc13 is degraded. In agreement with this, our results support the idea that cytokinesis induction during a prolonged metaphase arrest requires CDK-mediated inhibition of PP2A-B55^Pab1^. Nevertheless, we also show that, even in the presence of high CDK activity, PP2A-B55^Pab1^ together with PP2A-B56^Par1^ and Clp1 can counteract CDK phosphorylation. How can we reconcile these two seemingly contradictory findings? One possibility would be that local inactivation of PP2A-B55^Pab1^ at the SPB, (where the SIN components localise) or at the division site could account for the induction of septation, while a separate pool of PP2A-B55^Pab1^ could still be active and able to dephosphorylate mitotic substrates. Still, we need a more thorough understanding of the mechanisms of phosphatase regulation to be able to respond to this question.

Finally, we show that a number of cytokinesis regulators accumulate in the cell as the arrest persists. Whereas this could be the direct consequence of impaired degradation due to an inactive APC/C, we show that mitotic transcription also contributes to this phenomenon and the induction of septation in our system. In particular, mutation of the RFX transcription factor Sak1, an activator of the M transcriptional wave (Garg et al., 2015), impairs cytokinesis. Downstream of Sak1, the transcription factor Ace2 controls the expression of genes required for cell separation (*eng1, agn1, etc*.) and, as mentioned above, of *par2*. In addition, we show that deletion of *par1* hinders the expression of these genes. Banyai et al. (2016) have shown that cell cycle-regulated expression of the different transcriptional clusters responds to different thresholds of CDK activity. Hence, we thought that the incomplete induction of these genes in the *par1*Δ background was secondary to the lower CDK activity of this mutant. Nevertheless, addition of the *cdc2-3w* allele did not ameliorate this defect, indicating that PP2A-B56^Par1^ plays an additional role in promoting cytokinesis facilitating mitotic transcription.

**FIGURE 8:**
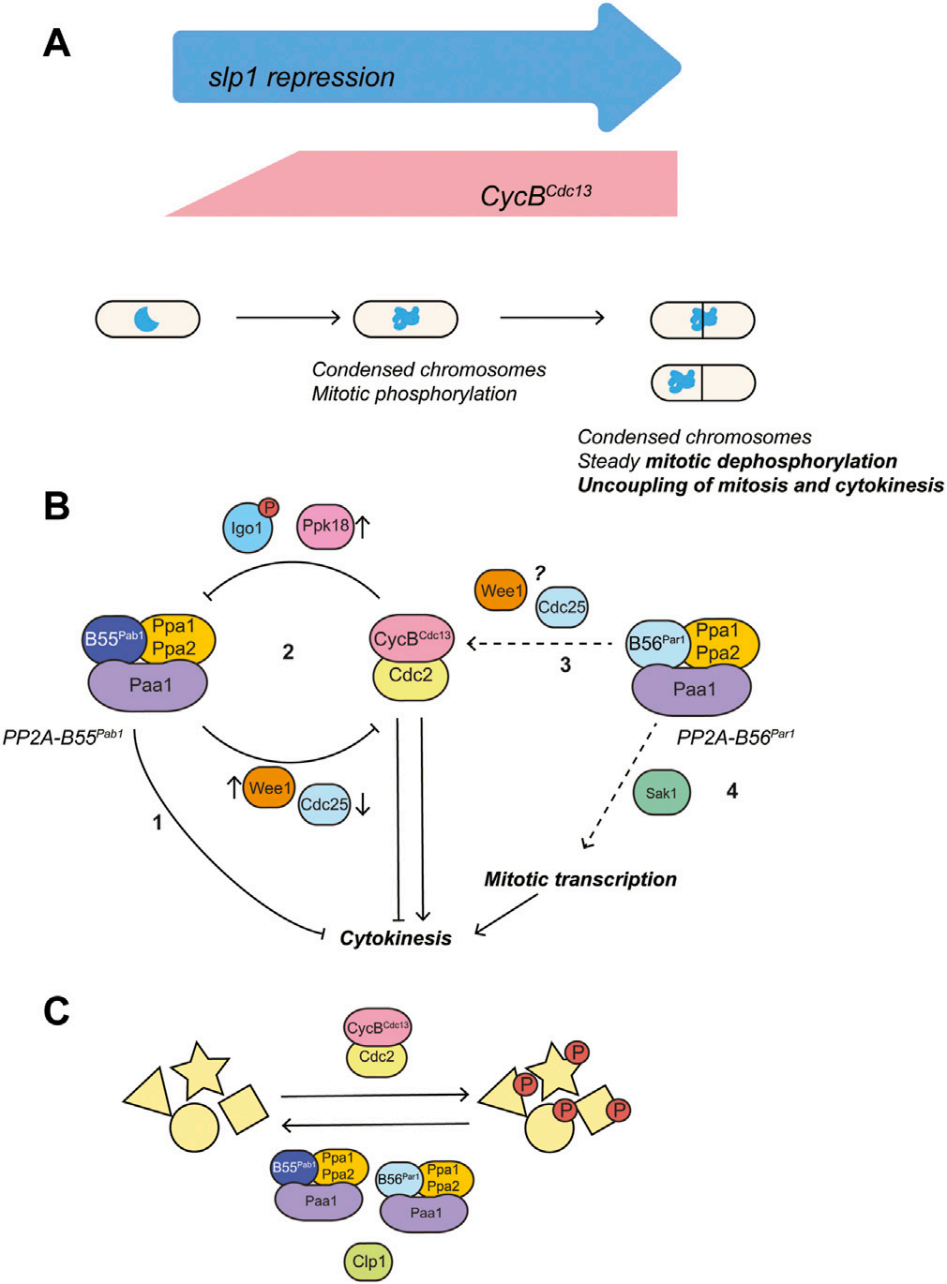
Proposed model for the regulation of cytokinesis by PP2A phosphatases and mitotic CDK complex during a prolonged mitotic arrest. **(A)** Mitotic arrest was induced through transcriptional repression of the APC/C activator Slp1 (under the control of the thiamine-repressible promoter *p41-nmt1*). Silencing of *slp1* resulted in the rapid accumulation of the mitotic cyclin Cdc13, increased mitotic phosphorylation and chromosome condensation. Upon prolonged periods of mitotic arrest, mitotic dephosphorylation and induction of cytokinesis could be observed in the presence of condensed chromosomes and high levels of Cdc13. **(B)** Mitotic CDK both favors and represses cytokinesis by inactivating the Spg1 GAP (activation of the top of the SIN cascade), while preventing recruitment of Sid1:Cdc14 (repression of the bottom of the SIN cascade) (Guertin et al., 2000; Johnson and Gould, 2011; Rachfall et al., 2014). PP2A-B55^Pab1^ and PP2A-B56^Par1^ have opposite effects in the regulation of cytokinesis: PP2A-B55^Pab1^ represses cytokinesis and, in its absence, untimely septation in the presence of unsegregated chromosomes occurs at high frequency in mitotically-arrested cells (1). The mitotic CDK complex establishes a double negative feedback loop with PP2A-B55^Pab1^ through the Ppk18-Igo1 (Greatwall-ENSA) pathway (2). In consequence, high CDK activity during a prolonged mitotic arrest would result in PP2A-B55^Pab1^ inactivation and induction of cytokinesis. In our experimental conditions, engagement of this double negative feedback loop would be sufficient to overcome the repression of cytokinesis by CDK. PP2A-B56^Par1^ favors cytokinesis through different means: it participates in the activation of the mitotic CDK complex at the G2/M transition (3). In its absence, cells enter mitosis with residual Cdc2-Tyr15 phosphorylation. Presumably, this hinders the repression of PP2A-B55^Pab1^ by CDK and results in septation being prevented. The underlying mechanism of this regulation is unknown at present, but might involve modulation of Wee1 and/or Cdc25 activities. In addition, PP2A-B56^Par1^ participates in the activation of the mitotic transcriptional program brought about by the RFX transcription factor Sak1 (4). Loss of either Sak1 or PP2A-B56^Par1^ impairs the expression of cytokinetic regulators and this contributes to the absence of cytokinesis during the arrest in these mutants. **(C)** Steady mitotic dephosphorylation during the arrest requires the activities of Clp1, PP2A-B55^Pab1^ and PP2A-B56^Par1^.

In summary, we have shown that fission yeast cells try to escape a prolonged mitotic arrest by inducing septation and gradually dephosphorylating mitotic substrates (Figure 8A). Protein phosphatases, particularly members of the PP2A family contribute to these events in different and complementary ways. On the one hand, PP2A-B55^Pab1^ inhibition induces cytokinesis, presumably by bypassing the SIN. In contrast, loss of PP2A-B56^Par1^ represses septation, and we track this phenotype down to a defect in CDK activation and disruption of the feedback loops connecting CDK and PP2A-B55^Pab1^ (Figure 8B). Mitotic dephosphorylation on the other hand requires the concerted action of both phosphatases and of Clp1 (Figure 8C). Finally, we show a prominent role for mitotic transcription in the escape from the arrest (Figure 8B). While some of the components influencing the behavior of our system are specific to fission yeast, others are key cell cycle regulators conserved across species. Seeing the striking similarities between mitotic escape in fission yeast and mitotic slippage in higher eukaryotes, we expect that the mechanisms here described are also relevant in these more complex systems.

## Experimental Procedures

### Yeast Strains and Growth Conditions

The fission yeast strains used in this study are listed in Supplementary Table S6. The cell culture and genetic manipulation of fission yeast strains were performed according to standard protocols (Moreno et al., 1991). All strains used were prototroph and were grown on Edinburgh minimal medium (EMM) containing NH_4_Cl 93.5mM as the source of nitrogen without supplements at 30°C in liquid or on solid media. For the repression of genes from the thiamine repressible promoter (*p41-nmt1*) and degradation of proteins tagged with an auxin inducible degron (miniAID version), cells grown to mid-exponential phase (4–6 × 10^6^ cells/ml) were shifted to EMM containing 15 µM of thiamine and/or 0.5 mM of NAA (1-Naphthaleneacetic acid), respectively. For Nda3 inactivation experiments using the temperature-sensitive allele *nda3-KM311*, cells were grown in EMM at 30°C and shifted to 18°C for 6 h. For the temperature sensitive *sak1* ^*ts*^ allele, cells were grown in EMM at 25°C and then shifted to 36°C for the time points indicated in the experiments.

### Fluorescent and Confocal Microscopy and Counting of Mitosis and Cell Division Stages

For fluorescent and confocal imaging, 200 µl of cells in the exponential phase were filtered and fixed with 200 µl of 70% ethanol. For permeabilization and staining, the cells were washed with PBS pH 7.2 and resuspended in a mix of 1.5 µl of 0,1 μg/ml DAPI (Sigma-Aldrich), 2 µl of 50 μg/ml of blankophor (Bayer) and 1.5 µl of PBS pH 7.2. Fluorescent imaging was carried out using a Zeiss Axio Observer fluorescent microscope equipped with a DAPI-specific filter and an objective Zeiss Plan-Apochromat ×100/1.40 Oil. The images were captured with an AxioCam MR R3 cooled CD camera controlled by the Zen software (Carl Zeiss). The counting of mitosis and cell division stages was carried out in more than 200 cells per each time point using Fiji software (http://fiji.sc/). Confocal imaging was carried out using a Zeiss LSM 710 Confocal microscope equipped with a Diode laser (405 nm) to excite DAPI and He-Ne laser (543/561 nm) to excite mCherry filter and an objective Zeiss Plan-Apochromat ×63/1.4 oil. The z-stacks images were acquired throughout each slide in a uniform fashion using a range of 4.5 µm and with an optimal range of 0.45 µm within each slice. Representative images were chosen.

### Genetic Manipulation and *nmt41* Strain Constructions

Gene deletion, promoter exchange, and gene tagging were carried out using PCR cassettes amplified from pFA6a derivative plasmids (Bähler et al., 1998). Gene deletion and double mutants were constructed by integration of cassettes in a wild-type background and subsequent genetic cross and tetrad dissection.

### Protein Extraction, Immunoblotting and Antibodies

For western blotting of fission yeast cells, 1 × 10^8^ cells were collected by filtration, washed with STOP buffer (150 mM NaCl, 50 mM NaF, 10 mM EDTA, 1 mM sodium azide) and frozen with liquid nitrogen before extraction. Total protein extracts were prepared by precipitation with trichloroacetic acid (TCA) as previously described (Foiani et al., 1994). Following gel electrophoresis in 4–20% Criterion™ TGX™ Precast Midi (BioRad), proteins were transferred onto a PVDF membrane using a semi-dry blotting system (Trans-Blot^®^ Turbo™ from BioRad). Membranes were blocked in a 5% skimmed milk solution in TBS-0.1% Tween and antibody solutions were prepared in TBS-Tween (0.1%) containing 5% BSA (for antibodies against phosphorylated residues) or 5% non-fat milk (in all other instances). Western blots were developed using Amersham ECL western blotting detection reagents and imaged either by x-ray film or Chemidoc MP Imaging system (Bio-Rad laboratories).

The following primary antibodies were used in this study: mouse anti-phospho-(Thr-Pro-101) to detect Cdk substrate-specific phosphorylations (1:1,000, Cell Signalling Technology), rabbit anti-phospho-cdc2 (Tyr15) to detect Cdc2-Y15-P (1:1,000, Cell Signalling Technology), anti-Cdc13 antibody (6F11/2) (1:1,000, Abcam), anti-Cdc2 (PSTAIR) (1:1,000, Millipore Sigma), anti-Byr4 (a kind gift from Prof. Kathleen Gould, 1:5,000). As secondary antibodies, we used HRP-conjugated anti mouse IgG (SIGMA, 1:10,000) and HRP-conjugated anti rabbit IgG (SIGMA, 1:10,000).

### FACS Analysis

For FACS analysis, 10^6^ cells were fixed in 70% ethanol and stained with propidium iodide according to published protocols (Sabatinos and Forsburg, 2009). The DNA content was determined by forward scatter analysis using FACSCalibur instrument and the data was analyzed using FlowJo software (Tree Star Inc., Ashland, OR, United States).

### RNA Extraction and Quantitative PCR (qPCR)

For qPCR, 5 × 10^7^ cells were collected by centrifugation, washed in DEPC-treated water and frozen with liquid nitrogen. Total RNA preparation was performed with MasterPure™ Yeast RNA Purification Kit (Epicentre) following the manufacturer instructions. 1 µg of RNA was used for cDNA synthesis using SuperScript^®^ III Reverse Transcriptase (Invitrogen). qPCR was performed with SYBR^®^ Select Master Mix (Applied Biosystems). Analysis was done using the ΔΔCt method. Oligos are listed in Supplementary Table S7.

### Sample Collection and Preparation for MS-Based Proteomic Analysis

All experiments were performed using label-free quantitative proteomics. Cultures were first grown to mid-exponential phase (5 × 10^6^ cells/ml) and were then diluted to 2.5 × 10^6^ cells/ml and shifted to EMM containing 15 µM of thiamine for 6 h for inducing *p41-nmt1:slp1* repression. The design of the experiment is detailed in Figure 6A. For sampling, 2.5 × 10^8^ cells were collected by filtration, first washed with 100 ml of PBS pH 7.2 and then washed with 1 ml of STOP buffer. Pellets were frozen in liquid nitrogen. For protein extraction, cell pellets were resuspended in denaturing lysis buffer containing 8 M urea, protease inhibitors (cOmplete™ Mini, Sigma-Aldrich) and phosphatase inhibitors (2 mM sodium pyrophosphate, 2 mM NaF, 2 mM β-GP and 2 mM sodium orthovanadate). Cell disruption was performed by agitation and homogenization with a cryogenic bead beating grinder (FastPrep®-24 5G). After centrifugation, 250 U of Benzonase^®^ (Sigma-Aldrich) were added to the protein extracts to remove nucleic acids and incubated for 10 min on ice. Protein concentration was determined by Bradford assay (Bio-Rad) (Casado et al., 2013). 400 ug of total protein extract was reduced with 10 mM dithiothreitol (DTT) for 30 min at 37°C and prepared for peptide digestion after alkylation with 10 mM iodoacetamide and reduction of the urea concentration to 2 M using the FASP™ protein digestion kit and following the manufacturer instructions (Expedeon). Proteins were digested using trypsin in a ratio 1:100 w/w (Promega-V511) dissolved in 50 mM acetic acid, 50 mM ammonium bicarbonate for 18 h at 37°C. Digested peptides were then acidified to 0.5% trifluoroacetic acid (TFA) and then desalted with 0.1% TFA, 5% acetonitrile (ACN) using Pierce® C18 tips-100 µl bed that were previously conditioned and equilibrated according to instructions (Thermo Scientific). The eluted peptides were dried under vacuum.

### LC–MS/MS Analysis

Peptide identification and abundance measurements were performed on an Orbitrap Elite mass spectrometer coupled to an Ultimate 3000 Rapid Separation LC system (both Thermo Scientific). Peptide mixtures were re-suspended in 14 µl 0.1% Formic acid (FA), 2% Acetonitrile (ACN), and injected twice (6 µl per injection). Each run consisted of a 4-h elution gradient with two mobile phases (solvent A: 0.1% FA in water - solvent B: 0.1% FA in ACN). The eluting peptides from the LC-column (nC18, 75 μm × 50 cm, 2 µm particle size) were ionized in the electrospray and analyzed by the Orbitrap Elite using Collision Induced Dissociation (CID) as activation method.

### Proteomics Data Structure, Processing, and Analysis

The raw mass spectrometry data was processed in Max Quant (Version 1.5.8.3) using default parameters including match between runs for peptide identification (Cox and Mann, 2008). The mapping of the proteins was searched against a Uniprot *S. pombe* proteome fasta file and corrected to discard common contaminant sequences using a FDR < 0.01 and a decoy revert mode for identification of reverse sequences. Maxquant output file was processed using Perseus (Version 1.6.12.01) (Tyanova et al., 2016) and after removal reverse and contaminant peptides, the LFQ intensities with >0 valid values were transformed into log2 scale, and then the repeated measurements were grouped into biological replicates and subsequently used for the analysis described in Figure 6B. Briefly, only protein values with ≥1 valid measurement per biological replicate for all conditions were used to perform a multiple-way ANOVA to find proteins with significant variation across all conditions. *p* values from the ANOVA were subjected to a permutation-based FDR correction. Supplementary Table S1 contains all significant proteins over 0.05 FDR cut-off. The proteins below to 0.001 FDR cut-off were used for hierarchical clustering analysis using the R package heatmap.2. Hierarchical clustering was performed on the log2 fold difference values from EMM t = 0 h using as settings cluster rows, Euclidian distance and ward.D2. The tree was cut into six groups.

In parallel, only proteins with ≥3 valid measurements in at least one replicate for all conditions were used to identify other proteins that were not consider in the first analysis. Differential expression analysis and a PCA were performed on the resultant protein list after imputation of missing values using Perseus (Supplementary Table S2). The proteins with significant differential expression between one thiamine-treated and one untreated sample were identified using a two-tailed test and plotted in the form of a volcano plot. Supplementary Table S2 also contains the significant hits from all differential analysis with permutation-based FDR adjusted *p*-values under a cut-off of 0.05. For the PCA, a multiple-way ANOVA was performed to identify the proteins with significant variation using 0.05 FDR cut-off (Supplementary Table S3).

### GO Enrichment Analysis, Annotations, and PPI Network Analysis

GO enrichment analysis was performed using Metascape (Zhou et al., 2019). Default Metascape parameters including a FDR < 0.01 were used with the following adjustments: GO BP, MF and CC were selected and Reactome and KEGG pathway unselected. For enrichment of clusters (Figure 6D), the analysis was performed using Supplementary Table S1 as a background dataset (*n* = 1,850) and for the enrichment of the significant upregulated proteins (≤0.05 FDR cut-off) in all differential expression analysis, Supplementary Table S2 was used as background dataset (*n* = 2,526). All significantly enriched GO categories are listed in Supplementary Tables S4, S5. GO annotations for volcano scatter plots were extracted from default annotations in Perseus version 1.6.12.01.

The PPI network analysis was performed in Metascape using MCODE algorithm to detect connectivity of the data based on known protein complexes (Bader and Hogue, 2003) and using *S. pombe* as input organism and min. size = 3 and max. size = 500 as settings of the network. Cytoscape was used to improve visualization and to track annotations based on adjacent words in the networks (Shannon et al., 2003).

### Quantifications and Statistical Analysis for Other Methods

To determine the ratio of CDK-P-(T/P)/Cdc2, representative WB raw images from x-ray film (Figures 1–3) and from digital ChemiDoc images (Figures 4, 5) were analyzed to calculate the densitometric values of the bands using Image lab software (Bio-Rad laboratories). Any given ratio was normalized to the untreated control sample in each experiment. For qPCR, all experiments were performed independently at least three times and the data were presented as mean ± SEM. Two-way ANOVA with Tukey’s multiple comparisons test was used to calculate significance differences in the qPCR analysis using GraphPad Prims v 8.0. Venn Diagram was performed in R.

## Data Availability

The mass spectrometry proteomics data have been deposited to the ProteomeXchange Consortium via the PRIDE [1] partner repository with the dataset identifier PXD031975 (Deutsch et al., 2017).

## References

[B1] Alcaide-Gavilán M. Lahoz A. Daga R. R. Jimenez J. (2014). Feedback Regulation of SIN by Etd1 and Rho1 in Fission Yeast. Genetics 196, 455–470. 10.1534/genetics.113.155218 24336750 PMC3914619

[B2] Aligue R. Wu L. Russell P. (1997). Regulation of *Schizosaccharomyces pombe* Wee1 Tyrosine Kinase. J. Biol. Chem. 272, 13320–13325. 10.1074/jbc.272.20.13320 9148953

[B3] Azzam R. Chen S. L. Shou W. Mah A. S. Alexandru G. Nasmyth K. (2004). Phosphorylation by Cyclin B-Cdk Underlies Release of Mitotic Exit Activator Cdc14 from the Nucleolus. Science 305, 516–519. 10.1126/science.1099402 15273393

[B4] Bähler J. Wu J.-Q. Longtine M. S. Shah N. G. Mckenzie III A. Steever A. B. (1998). Heterologous Modules for Efficient and Versatile PCR-Based Gene Targeting in *Schizosaccharomyces pombe* . Yeast 14, 943–951. 10.1002/(sici)1097-0061(199807)14:10<943::aid-yea292>3.0.co;2-y 9717240

[B5] Bader G. D. Hogue C. W. (2003). An Automated Method for Finding Molecular Complexes in Large Protein Interaction Networks. BMC Bioinforma. 4, 2. 10.1186/1471-2105-4-2 PMC149346 12525261

[B6] Banyai G. Baïdi F. Coudreuse D. Szilagyi Z. (2016). Cdk1 Activity Acts as a Quantitative Platform for Coordinating Cell Cycle Progression with Periodic Transcription. Nat. Commun. 7, 11161. 10.1038/ncomms11161 27045731 PMC4822045

[B7] Basu S. Roberts E. L. Jones A. W. Swaffer M. P. Snijders A. P. Nurse P. (2020). The Hydrophobic Patch Directs Cyclin B to Centrosomes to Promote Global CDK Phosphorylation at Mitosis. Curr. Biol. 30, 883–892.e4. 10.1016/j.cub.2019.12.053 32084401 PMC7063568

[B8] Bouchoux C. Uhlmann F. (2011). A Quantitative Model for Ordered Cdk Substrate Dephosphorylation during Mitotic Exit. Cell 147, 803–814. 10.1016/j.cell.2011.09.047 22078879

[B9] Brito D. A. Rieder C. L. (2006). Mitotic Checkpoint Slippage in Humans Occurs via Cyclin B Destruction in the Presence of an Active Checkpoint. Curr. Biol. 16, 1194–1200. 10.1016/j.cub.2006.04.043 16782009 PMC2749311

[B10] Burgess A. Vigneron S. Brioudes E. Labbé J.-C. Lorca T. Castro A. (2010). Loss of Human Greatwall Results in G2 Arrest and Multiple Mitotic Defects Due to Deregulation of the Cyclin B-Cdc2/PP2A Balance. Proc. Natl. Acad. Sci. U.S.A. 107, 12564–12569. 10.1073/pnas.0914191107 20538976 PMC2906566

[B11] Casado P. Rodriguez-Prados J.-C. Cosulich S. C. Guichard S. Vanhaesebroeck B. Joel S. (2013). Kinase-substrate Enrichment Analysis Provides Insights into the Heterogeneity of Signaling Pathway Activation in Leukemia Cells. Sci. Signal. 6, rs6. 10.1126/scisignal.2003573 23532336

[B12] Cerutti L. Simanis V. (1999). Asymmetry of the Spindle Pole Bodies and Spg1p GAP Segregation during Mitosis in Fission Yeast. J. Cell Sci. 112 (Pt 14), 2313–2321. 10.1242/jcs.112.14.2313 10381387

[B13] Chang L. Morrell J. L. Feoktistova A. Gould K. L. (2001). Study of Cyclin Proteolysis in Anaphase-Promoting Complex (APC) Mutant Cells Reveals the Requirement for APC Function in the Final Steps of the Fission Yeast Septation Initiation Network. Mol. Cell Biol. 21, 6681–6694. 10.1128/MCB.21.19.6681-6694.2001 11533255 PMC99813

[B14] Chen C.-T. Feoktistova A. Chen J.-S. Shim Y.-S. Clifford D. M. Gould K. L. (2008). The SIN Kinase Sid2 Regulates Cytoplasmic Retention of the *S. pombe* Cdc14-like Phosphatase Clp1. Curr. Biol. 18, 1594–1599. 10.1016/j.cub.2008.08.067 18951025 PMC2824331

[B15] Chica N. Rozalén A. E. Pérez-Hidalgo L. Rubio A. Novak B. Moreno S. (2016). Nutritional Control of Cell Size by the Greatwall-Endosulfine-PP2A·B55 Pathway. Curr. Biol. 26, 319–330. 10.1016/j.cub.2015.12.035 26776736

[B16] Cox J. Mann M. (2008). MaxQuant Enables High Peptide Identification Rates, Individualized p.p.b.-range Mass Accuracies and Proteome-wide Protein Quantification. Nat. Biotechnol. 26, 1367–1372. 10.1038/nbt.1511 19029910

[B17] Csikász-Nagy A. Kapuy O. Győrffy B. Tyson J. J. Novák B. (2007). Modeling the Septation Initiation Network (SIN) in Fission Yeast Cells. Curr. Genet. 51, 245–255. 10.1007/s00294-007-0123-4 17340144

[B18] Cueille N. Salimova E. Esteban V. Blanco M. Moreno S. Bueno A. (2001). Flp1, a Fission Yeast Orthologue of the s. cerevisiae CDC14 Gene, Is Not Required for Cyclin Degradation or Rum1p Stabilisation at the End of Mitosis. J. Cell Sci. 114, 2649–2664. 10.1242/jcs.114.14.2649 11683392

[B19] Daga R. R. Lahoz A. Muñoz M. J. Moreno S. Jimenez J. (2005). Etd1p Is a Novel Protein that Links the SIN Cascade with Cytokinesis. Embo J. 24, 2436–2446. 10.1038/sj.emboj.7600705 15933715 PMC1173146

[B100] Deutsch E. W. Csordas A. Sun Z. Jarnuczak A. Perez-Riverol Y. Ternent T. (2017). The Proteomexchange Consortium In 2017: Supporting the Cultural Change in Proteomics Public Data Deposition. Nucleic Acids Res. 45 (Database issue), D1100–D1106. 27924013 10.1093/nar/gkw936 PMC5210636

[B20] Enoch T. Carr A. M. Nurse P. (1992). Fission Yeast Genes Involved in Coupling Mitosis to Completion of DNA Replication. Genes Dev. 6, 2035–2046. 10.1101/gad.6.11.2035 1427071

[B21] Esteban V. Blanco M. Cueille N. Simanis V. Moreno S. Bueno A. (2004). A Role for the Cdc14-Family Phosphatase Flp1p at the End of the Cell Cycle in Controlling the Rapid Degradation of the Mitotic Inducer Cdc25p in Fission Yeast. J. Cell Sci. 117, 2461–2468. 10.1242/jcs.01107 15128870

[B22] Fantes P. A. (1981). Isolation of Cell Size Mutants of a Fission Yeast by a New Selective Method: Characterization of Mutants and Implications for Division Control Mechanisms. J. Bacteriol. 146, 746–754. 10.1128/jb.146.2.746-754.1981 7217015 PMC217021

[B23] Foiani M. Marini F. Gamba D. Lucchini G. Plevani P. (1994). The B Subunit of the DNA Polymerase Alpha-Primase Complex in *Saccharomyces cerevisiae* Executes an Essential Function at the Initial Stage of DNA Replication. Mol. Cell. Biol. 14, 923–933. 10.1128/mcb.14.2.923-933.1994 8289832 PMC358447

[B24] Funabiki H. Yamano H. Nagao K. Tanaka H. Yasuda H. Hunt T. (1997). Fission Yeast Cut2 Required for Anaphase Has Two Destruction Boxes. EMBO J. 16, 5977–5987. 10.1093/emboj/16.19.5977 9312055 PMC1170228

[B25] Furge K. A. Wong K. Armstrong J. Balasubramanian M. Albright C. F. (1998). Byr4 and Cdc16 Form a Two-Component GTPase-Activating Protein for the Spg1 GTPase that Controls Septation in Fission Yeast. Curr. Biol. 8, 947–954. 10.1016/S0960-9822(98)70394-X 9742395

[B26] Garg A. Futcher B. Leatherwood J. (2015). A New Transcription Factor for Mitosis: in *Schizosaccharomyces pombe*, the RFX Transcription Factor Sak1 Works with Forkhead Factors to Regulate Mitotic Expression. Nucleic Acids Res. 43, 6874–6888. 10.1093/nar/gkv274 25908789 PMC4538799

[B27] Gharbi-Ayachi A. Labbé J.-C. Burgess A. Vigneron S. Strub J.-M. Brioudes E. (2010). The Substrate of Greatwall Kinase, Arpp19, Controls Mitosis by Inhibiting Protein Phosphatase 2A. Science 330, 1673–1677. 10.1126/science.1197048 21164014

[B28] Goyal A. Simanis V. (2012). Characterization of Ypa1 and Ypa2, the *Schizosaccharomyces pombe* Orthologs of the Peptidyl Proyl Isomerases that Activate PP2A, Reveals a Role for Ypa2p in the Regulation of Cytokinesis. Genetics 190, 1235–1250. 10.1534/genetics.111.138040 22267499 PMC3316640

[B29] Grallert A. Boke E. Hagting A. Hodgson B. Connolly Y. Griffiths J. R. (2015). A PP1-PP2A Phosphatase Relay Controls Mitotic Progression. Nature 517, 94–98. 10.1038/nature14019 25487150 PMC4338534

[B30] Guertin D. A. Chang L. Irshad F. Gould K. L. McCollum D. (2000). The Role of the Sid1p Kinase and Cdc14p in Regulating the Onset of Cytokinesis in Fission Yeast. EMBO J. 19, 1803–1815. 10.1093/emboj/19.8.1803 10775265 PMC302011

[B31] Hanahan D. Weinberg R. A. (2011). Hallmarks of Cancer: The Next Generation. Cell 144, 646–674. 10.1016/j.cell.2011.02.013 21376230

[B32] He X. Patterson T. E. Sazer S. (1997). The *Schizosaccharomyces pombe* Spindle Checkpoint Protein Mad2p Blocks Anaphase and Genetically Interacts with the Anaphase-Promoting Complex. Proc. Natl. Acad. Sci. U.S.A. 94, 7965–7970. 10.1073/pnas.94.15.7965 9223296 PMC21538

[B33] He X. Jones M. H. Winey M. Sazer S. (1998). Mph1, a Member of the Mps1-like Family of Dual Specificity Protein Kinases, Is Required for the Spindle Checkpoint in *S. pombe* . J. Cell Sci. 111 (Pt 12), 1635–1647. 10.1242/jcs.111.12.1635 9601094

[B34] Hirano T. Funahashi S.-i. Uemura T. Yanagida M. (1986). Isolation and Characterization of *Schizosaccharomyces pombe* Cut Mutants that Block Nuclear Division but Not Cytokinesis. EMBO J. 5, 2973–2979. 10.1002/j.1460-2075.1986.tb04594.x 16453724 PMC1167249

[B35] Hiraoka Y. Toda T. Yanagida M. (1984). The NDA3 Gene of Fission Yeast Encodes β-tubulin: A Cold-Sensitive Nda3 Mutation Reversibly Blocks Spindle Formation and Chromosome Movement in Mitosis. Cell 39, 349–358. 10.1016/0092-8674(84)90013-8 6094012

[B36] Hocquet C. Robellet X. Modolo L. Sun X.-M. Burny C. Cuylen-Haering S. (2018). Condensin Controls Cellular RNA Levels through the Accurate Segregation of Chromosomes Instead of Directly Regulating Transcription. eLife 7, e38517. 10.7554/eLife.38517 30230473 PMC6173581

[B37] Janssens V. Longin S. Goris J. (2008). PP2A Holoenzyme Assembly: in Cauda Venenum (The Sting Is in the Tail). Trends Biochem. Sci. 33, 113–121. 10.1016/j.tibs.2007.12.004 18291659

[B38] Jiang W. Hallberg R. L. (2000). Isolation and Characterization of Par1(+) and Par2(+): Two *Schizosaccharomyces pombe* Genes Encoding B’ Subunits of Protein Phosphatase 2A. Genetics 154, 1025–1038. 10.1093/genetics/154.3.1025 10757751 PMC1460981

[B39] Johnson A. E. Gould K. L. (2011). Dma1 Ubiquitinates the SIN Scaffold, Sid4, to Impede the Mitotic Localization of Plo1 Kinase. EMBO J. 30, 341–354. 10.1038/emboj.2010.317 21131906 PMC3025462

[B40] Johnson A. E. McCollum D. Gould K. L. (2012). Polar Opposites: Fine-tuning Cytokinesis through SIN Asymmetry. Cytoskeleton 69, 686–699. 10.1002/cm.21044 22786806 PMC3478943

[B41] Kakui Y. Rabinowitz A. Barry D. J. Uhlmann F. (2017). Condensin-mediated Remodeling of the Mitotic Chromatin Landscape in Fission Yeast. Nat. Genet. 49, 1553–1557. 10.1038/ng.3938 28825727 PMC5621628

[B42] Kim S. H. Lin D. P. Matsumoto S. Kitazono A. Matsumoto T. (1998). Fission Yeast Slp1: an Effector of the Mad2-dependent Spindle Checkpoint. Science 279, 1045–1047. 10.1126/science.279.5353.1045 9461438

[B43] Kinoshita N. Yamano H. Niwa H. Yoshida T. Yanagida M. (1993). Negative Regulation of Mitosis by the Fission Yeast Protein Phosphatase Ppa2. Genes Dev. 7, 1059–1071. 10.1101/gad.7.6.1059 8389306

[B44] Kovelman R. Russell P. (1996). Stockpiling of Cdc25 during a DNA Replication Checkpoint Arrest in *Schizosaccharomyces pombe* . Mol. Cell Biol. 16, 86–93. 10.1128/MCB.16.1.86 8524332 PMC230981

[B45] Krapp A. Simanis V. (2008). An Overview of the Fission Yeast Septation Initiation Network (SIN). Biochem. Soc. Trans. 36, 411–415. 10.1042/BST0360411 18481970

[B46] Krapp A. Cano E. Simanis V. (2003). Mitotic Hyperphosphorylation of the Fission Yeast SIN Scaffold Protein Cdc11p Is Regulated by the Protein Kinase Cdc7p. Curr. Biol. 13, 168–172. 10.1016/s0960-9822(02)01417-3 12546793

[B47] Lahoz A. Alcaide-Gavilán M. Daga R. R. Jimenez J. (2010). Antagonistic Roles of PP2A-Pab1 and Etd1 in the Control of Cytokinesis in Fission Yeast. Genetics 186, 1261–1270. 10.1534/genetics.110.121368 20876564 PMC2998309

[B48] Le Goff X. Buvelot S. Salimova E. Guerry F. Schmidt S. Cueille N. (2001). The Protein Phosphatase 2A B’-regulatory Subunit Par1p Is Implicated in Regulation of the *S. pombe* Septation Initiation Network. FEBS Lett. 508, 136–142. 10.1016/s0014-5793(01)03047-2 11707284

[B49] Lee J. Kim J. A. Margolis R. L. Fotedar R. (2010). Substrate Degradation by the Anaphase Promoting Complex Occurs during Mitotic Slippage. Cell Cycle 9, 1792–1801. 10.4161/cc.9.9.11519 20436289 PMC3163903

[B50] López-Avilés S. Kapuy O. Novák B. Uhlmann F. (2009). Irreversibility of Mitotic Exit Is the Consequence of Systems-Level Feedback. Nature 459, 592–595. 10.1038/nature07984 19387440 PMC2817895

[B51] Manchado E. Guillamot M. de Cárcer G. Eguren M. Trickey M. García-Higuera I. (2010). Targeting Mitotic Exit Leads to Tumor Regression *In Vivo*: Modulation by Cdk1, Mastl, and the PP2A/B55α,δ Phosphatase. Cancer Cell 18, 641–654. 10.1016/j.ccr.2010.10.028 21156286

[B52] Martín R. Portantier M. Chica N. Nyquist-Andersen M. Mata J. Lopez-Aviles S. (2017). A PP2A-B55-Mediated Crosstalk between TORC1 and TORC2 Regulates the Differentiation Response in Fission Yeast. Curr. Biol. 27, 175–188. 10.1016/j.cub.2016.11.037 28041796 PMC5266790

[B53] Martin S. G. Berthelot-Grosjean M. (2009). Polar Gradients of the DYRK-Family Kinase Pom1 Couple Cell Length with the Cell Cycle. Nature 459, 852–856. 10.1038/nature08054 19474792

[B54] Matsumoto T. (1997). A Fission Yeast Homolog of CDC20/p55CDC/Fizzy Is Required for Recovery from DNA Damage and Genetically Interacts with P34cdc2. Mol. Cell Biol. 17, 742–750. 10.1128/MCB.17.2.742 9001228 PMC231800

[B55] McInerny C. J. (2011). “2 - Cell Cycle Regulated Gene Expression in Yeasts,” in Advances in Genetics. Editors Friedmann T. Dunlap J. C. Goodwin S. F. (Academic Press), 51–85. 10.1016/B978-0-12-380860-8.00002-1 21310294

[B56] Mishra M. Karagiannis J. Sevugan M. Singh P. Balasubramanian M. K. (2005). The 14-3-3 Protein Rad24p Modulates Function of the Cdc14p Family Phosphatase Clp1p/flp1p in Fission Yeast. Curr. Biol. 15, 1376–1383. 10.1016/j.cub.2005.06.070 16085489

[B57] Mochida S. Ikeo S. Gannon J. Hunt T. (2009). Regulated Activity of PP2A-B55δ Is Crucial for Controlling Entry into and Exit from Mitosis in Xenopus Egg Extracts. EMBO J. 28, 2777–2785. 10.1038/emboj.2009.238 19696736 PMC2750019

[B58] Mochida S. Maslen S. L. Skehel M. Hunt T. (2010). Greatwall Phosphorylates an Inhibitor of Protein Phosphatase 2Α that Is Essential for Mitosis. Science 330, 1670–1673. 10.1126/science.1195689 21164013

[B59] Moreno S. Klar A. Nurse P. (1991). Molecular Genetic Analysis of Fission Yeast *Schizosaccharomyces pombe* . Methods Enzymol. 194, 795–823. 10.1016/0076-6879(91)94059-l 2005825

[B60] Morgan D. O. (2007). The Cell Cycle: Principles of Control. London: New science press.

[B61] Moseley J. B. Mayeux A. Paoletti A. Nurse P. (2009). A Spatial Gradient Coordinates Cell Size and Mitotic Entry in Fission Yeast. Nature 459, 857–860. 10.1038/nature08074 19474789

[B62] Murone M. Simanis V. (1996). The Fission Yeast Dma1 Gene Is a Component of the Spindle Assembly Checkpoint, Required to Prevent Septum Formation and Premature Exit from Mitosis if Spindle Function Is Compromised. EMBO J. 15, 6605–6616. 10.1002/j.1460-2075.1996.tb01051.x 8978687 PMC452485

[B63] Petrova B. Dehler S. Kruitwagen T. Hériché J.-K. Miura K. Haering C. H. (2013). Quantitative Analysis of Chromosome Condensation in Fission Yeast. Mol. Cell Biol. 33, 984–998. 10.1128/MCB.01400-12 23263988 PMC3623070

[B64] Queralt E. Lehane C. Novak B. Uhlmann F. (2006). Downregulation of PP2A(Cdc55) Phosphatase by Separase Initiates Mitotic Exit in Budding Yeast. Cell 125, 719–732. 10.1016/j.cell.2006.03.038 16713564

[B65] Rachfall N. Johnson A. E. Mehta S. Chen J.-S. Gould K. L. (2014). Cdk1 Promotes Cytokinesis in Fission Yeast through Activation of the Septation Initiation Network. MBoC 25, 2250–2259. 10.1091/mbc.E14-04-0936 24920823 PMC4116299

[B66] Rata S. Suarez Peredo Rodriguez M. F. Joseph S. Peter N. Echegaray Iturra F. Yang F. (2018). Two Interlinked Bistable Switches Govern Mitotic Control in Mammalian Cells. Curr. Biol. 28, 3824–3832.e6. 10.1016/j.cub.2018.09.059 30449668 PMC6287978

[B67] Sabatinos S. A. Forsburg S. L. (2009). Measuring DNA Content by Flow Cytometry in Fission Yeast. Methods Mol. Biol. 521, 449–461. 10.1007/978-1-60327-815-7_25 19563122

[B68] Sackton K. L. Dimova N. Zeng X. Tian W. Zhang M. Sackton T. B. (2014). Synergistic Blockade of Mitotic Exit by Two Chemical Inhibitors of the APC/C. Nature 514, 646–649. 10.1038/nature13660 25156254 PMC4214887

[B69] Samejima I. Matsumoto T. Nakaseko Y. Beach D. Yanagida M. (1993). Identification of Seven New Cut Genes Involved in *Schizosaccharomyces pombe* Mitosis. J. Cell Sci. 105 (Pt 1), 135–143. 10.1242/jcs.105.1.135 8395535

[B70] Schmidt S. Sohrmann M. Hofmann K. Woollard A. Simanis V. (1997). The Spg1p GTPase Is an Essential, Dosage-dependent Inducer of Septum Formation in *Schizosaccharomyces pombe* . Genes Dev. 11, 1519–1534. 10.1101/gad.11.12.1519 9203579

[B71] Shannon P. Markiel A. Ozier O. Baliga N. S. Wang J. T. Ramage D. (2003). Cytoscape: a Software Environment for Integrated Models of Biomolecular Interaction Networks. Genome Res. 13, 2498–2504. 10.1101/gr.1239303 14597658 PMC403769

[B72] Singh N. S. Shao N. McLean J. R. Sevugan M. Ren L. Chew T. G. (2011). SIN-Inhibitory Phosphatase Complex (SIP) Promotes Cdc11p Dephosphorylation and Propagates SIN Asymmetry in Fission Yeast. Curr. Biol. 21, 1968–1978. 10.1016/j.cub.2011.10.051 22119525 PMC4167312

[B73] Skoufias D. A. Indorato R.-L. Lacroix F. Panopoulos A. Margolis R. L. (2007). Mitosis Persists in the Absence of Cdk1 Activity when Proteolysis or Protein Phosphatase Activity Is Suppressed. J. Cell Biol. 179, 671–685. 10.1083/jcb.200704117 18025303 PMC2080905

[B74] Sloss O. Topham C. Diez M. Taylor S. (2016). Mcl-1 Dynamics Influence Mitotic Slippage and Death in Mitosis. Oncotarget 7, 5176–5192. 10.18632/oncotarget.6894 26769847 PMC4868679

[B75] Stonyte V. Martín R. Segura-Peña D. Sekulić N. Lopez-Aviles S. (2020). Requirement of PP2A-B56Par1 for the Stabilization of the CDK Inhibitor Rum1 and Activation of APC/CSte9 during Pre-Start G1 in *S. pombe* . iScience 23, 101063. 10.1016/j.isci.2020.101063 32361273 PMC7195536

[B76] Suárez M. B. Alonso-Nuñez M. L. del Rey F. McInerny C. J. Vázquez de Aldana C. R. (2015). Regulation of Ace2-dependent Genes Requires Components of the PBF Complex in *Schizosaccharomyces pombe* . Cell Cycle 14, 3124–3137. 10.1080/15384101.2015.1078035 26237280 PMC4825577

[B77] Sveiczer A. Csikasz-Nagy A. Gyorffy B. Tyson J. J. Novak B. (2000). Modeling the Fission Yeast Cell Cycle: Quantized Cycle Times in Wee1− Cdc25Δ Mutant Cells. Proc. Natl. Acad. Sci. U.S.A. 97, 7865–7870. 10.1073/pnas.97.14.7865 10884416 PMC16636

[B78] Topham C. H. Taylor S. S. (2013). Mitosis and Apoptosis: How Is the Balance Set? Curr. Opin. Cell Biol. 25, 780–785. 10.1016/j.ceb.2013.07.003 23890995

[B79] Touati S. A. Hofbauer L. Jones A. W. Snijders A. P. Kelly G. Uhlmann F. (2019). Cdc14 and PP2A Phosphatases Cooperate to Shape Phosphoproteome Dynamics during Mitotic Exit. Cell Rep. 29, 2105–2119.e4. 10.1016/j.celrep.2019.10.041 31722221 PMC6857435

[B80] Trautmann S. Wolfe B. A. Jorgensen P. Tyers M. Gould K. L. McCollum D. (2001). Fission Yeast Clp1p Phosphatase Regulates G2/M Transition and Coordination of Cytokinesis with Cell Cycle Progression. Curr. Biol. 11, 931–940. 10.1016/s0960-9822(01)00268-8 11448769

[B81] Tyanova S. Temu T. Cox J. (2016). The MaxQuant Computational Platform for Mass Spectrometry-Based Shotgun Proteomics. Nat. Protoc. 11, 2301–2319. 10.1038/nprot.2016.136 27809316

[B82] Umesono K. Hiraoka Y. Toda T. Yanagida M. (1983). Visualization of Chromosomes in Mitotically Arrested Cells of the Fission Yeast *Schizosaccharomyces pombe* . Curr. Genet. 7, 123–128. 10.1007/BF00365637 24173154

[B83] Uzawa S. Samejima I. Hirano T. Tanaka K. Yanagida M. (1990). The Fission Yeast Cut1+ Gene Regulates Spindle Pole Body Duplication and Has Homology to the Budding Yeast ESP1 Gene. Cell 62, 913–925. 10.1016/0092-8674(90)90266-h 2203537

[B84] Vigneron S. Brioudes E. Burgess A. Labbé J.-C. Lorca T. Castro A. (2009). Greatwall Maintains Mitosis through Regulation of PP2A. EMBO J. 28, 2786–2793. 10.1038/emboj.2009.228 19680222 PMC2750022

[B85] Visconti R. Della Monica R. Palazzo L. D'Alessio F. Raia M. Improta S. (2015). The Fcp1-Wee1-Cdk1 axis Affects Spindle Assembly Checkpoint Robustness and Sensitivity to Antimicrotubule Cancer Drugs. Cell Death Differ. 22, 1551–1560. 10.1038/cdd.2015.13 25744022 PMC4532778

[B86] Visintin R. Craig K. Hwang E. S. Prinz S. Tyers M. Amon A. (1998). The Phosphatase Cdc14 Triggers Mitotic Exit by Reversal of Cdk-dependent Phosphorylation. Mol. Cell 2, 709–718. 10.1016/s1097-2765(00)80286-5 9885559

[B87] Williams B. C. Filter J. J. Blake-Hodek K. A. Wadzinski B. E. Fuda N. J. Shalloway D. (2014). Greatwall-phosphorylated Endosulfine Is Both an Inhibitor and a Substrate of PP2A-B55 Heterotrimers. eLife 3, e01695. 10.7554/eLife.01695 24618897 PMC3949306

[B88] Wolfe B. A. Gould K. L. (2004). Fission Yeast Clp1p Phosphatase Affects G2/M Transition and Mitotic Exit through Cdc25p Inactivation. EMBO J. 23, 919–929. 10.1038/sj.emboj.7600103 14765109 PMC381010

[B99] Wolfe B. A. McDonald W. H. Yates J. R. Gould K. L. (2006). Phospho-Regulation of the Cdc14/Clp1 Phosphatase Delays Late Mitotic Events in *S. pombe* . Dev. Cell. 11 (3), 423–430. 10.1016/j.devcel.2006.07.016 16950131

[B89] Yamada H. Kumada K. Yanagida M. (1997). Distinct Subunit Functions and Cell Cycle Regulated Phosphorylation of 20S APC/cyclosome Required for Anaphase in Fission Yeast. J. Cell Sci. 110 (Pt 15), 1793–1804. 10.1242/jcs.110.15.1793 9264466

[B90] Yamano H. Gannon J. Hunt T. (1996). The Role of Proteolysis in Cell Cycle Progression in *Schizosaccharomyces pombe* . EMBO J. 15, 5268–5279. 10.1002/j.1460-2075.1996.tb00912.x 8895572 PMC452271

[B91] Yamashita Y. M. Nakaseko Y. Samejima I. Kumada K. Yamada H. Michaelson D. (1996). 20S Cyclosome Complex Formation and Proteolytic Activity Inhibited by the cAMP/PKA Pathway. Nature 384, 276–279. 10.1038/384276a0 8918880

[B92] Yamashita Y. M. Nakaseko Y. Kumada K. Nakagawa T. Yanagida M. (1999). Fission Yeast APC/cyclosome Subunits, Cut20/Apc4 and Cut23/Apc8, in Regulating Metaphase-Anaphase Progression and Cellular Stress Responses. Genes Cells 4, 445–463. 10.1046/j.1365-2443.1999.00274.x 10526233

[B93] Yanagida M. Yamashita Y. M. Tatebe H. Ishii K. Kumada K. Nakaseko Y. (1999). Control of Metaphase-Anaphase Progression by Proteolysis: Cyclosome Function Regulated by the Protein Kinase A Pathway, Ubiquitination and Localization. Phil. Trans. R. Soc. Lond. B 354, 1559–1569. discussion 1569-1570. 10.1098/rstb.1999.0499 10582241 PMC1692673

[B94] Yen H.-C. S. Gordon C. Chang E. C. (2003). *Schizosaccharomyces pombe* Int6 and Ras Homologs Regulate Cell Division and Mitotic Fidelity via the Proteasome. Cell 112, 207–217. 10.1016/s0092-8674(03)00043-6 12553909

[B95] Yuasa T. Hayashi T. Ikai N. Katayama T. Aoki K. Obara T. (2004). An Interactive Gene Network for Securin-Separase, Condensin, Cohesin, Dis1/Mtc1 and Histones Constructed by Mass Transformation. Genes Cells 9, 1069–1082. 10.1111/j.1365-2443.2004.00790.x 15507118

[B96] Yukawa M. Teratani Y. Toda T. (2021). Escape from Mitotic Catastrophe by Actin-dependent Nuclear Displacement in Fission Yeast. iScience 24, 102031. 10.1016/j.isci.2020.102031 33506191 PMC7814194

[B97] Zachariae W. Schwab M. Nasmyth K. Seufert W. (1998). Control of Cyclin Ubiquitination by CDK-Regulated Binding of Hct1 to the Anaphase Promoting Complex. Science 282, 1721–1724. 10.1126/science.282.5394.1721 9831566

[B98] Zhou Y. Zhou B. Pache L. Chang M. Khodabakhshi A. H. Tanaseichuk O. (2019). Metascape Provides a Biologist-Oriented Resource for the Analysis of Systems-Level Datasets. Nat. Commun. 10, 1523. 10.1038/s41467-019-09234-6 30944313 PMC6447622

