# Supplementary figures and images for: Uncoupling of Mitosis and Cytokinesis Upon a Prolonged Arrest in Metaphase Is Influenced by Protein Phosphatases and Mitotic Transcription in Fission Yeast
Source: Front Cell Dev Biol. 2022 Jul 18;10:876810. doi: 10.3389/fcell.2022.876810 (PMC9340479; doi:10.3389/fcell.2022.876810)

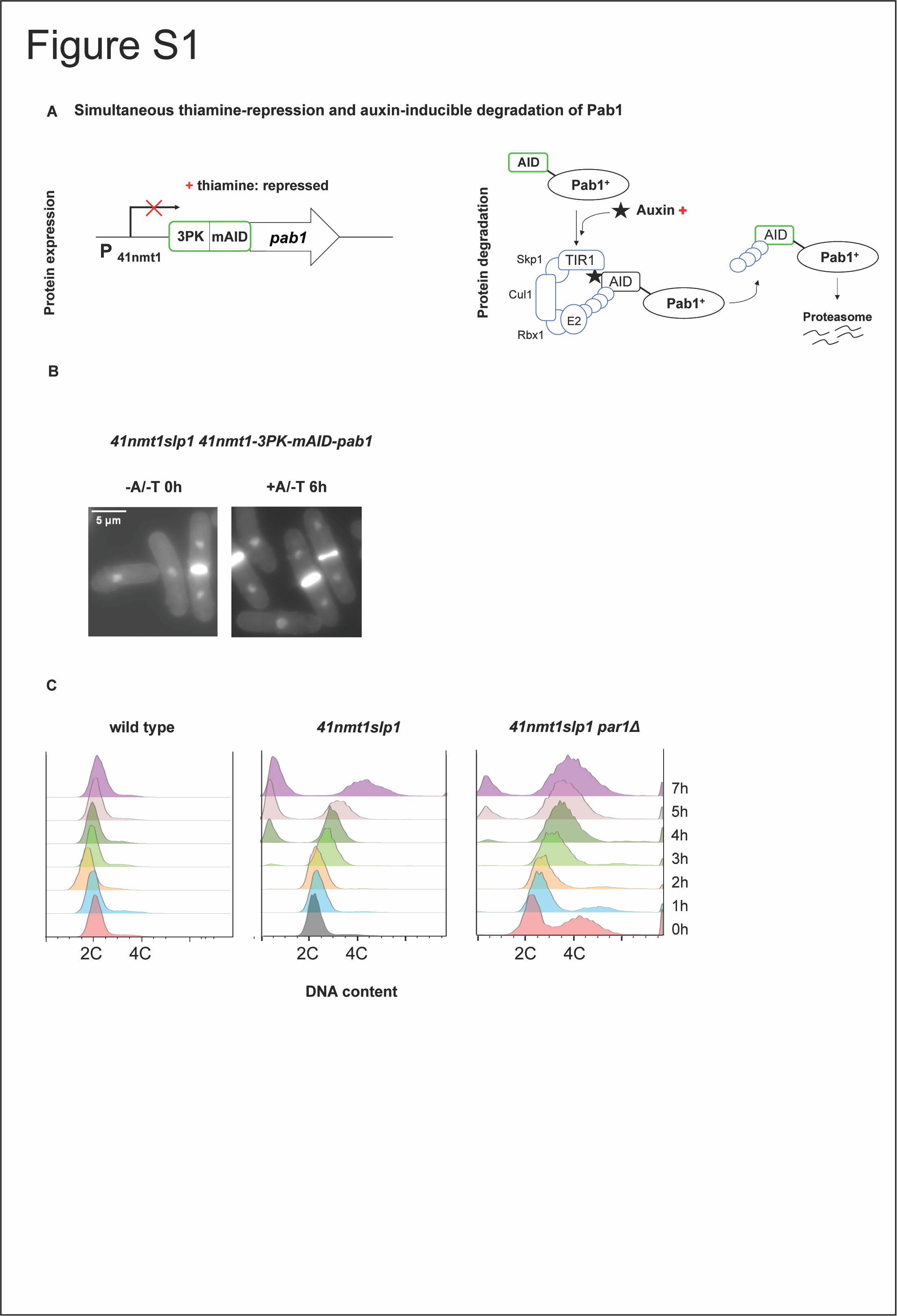

Supplement: Supplementary file 2 [file Image1.TIFF]

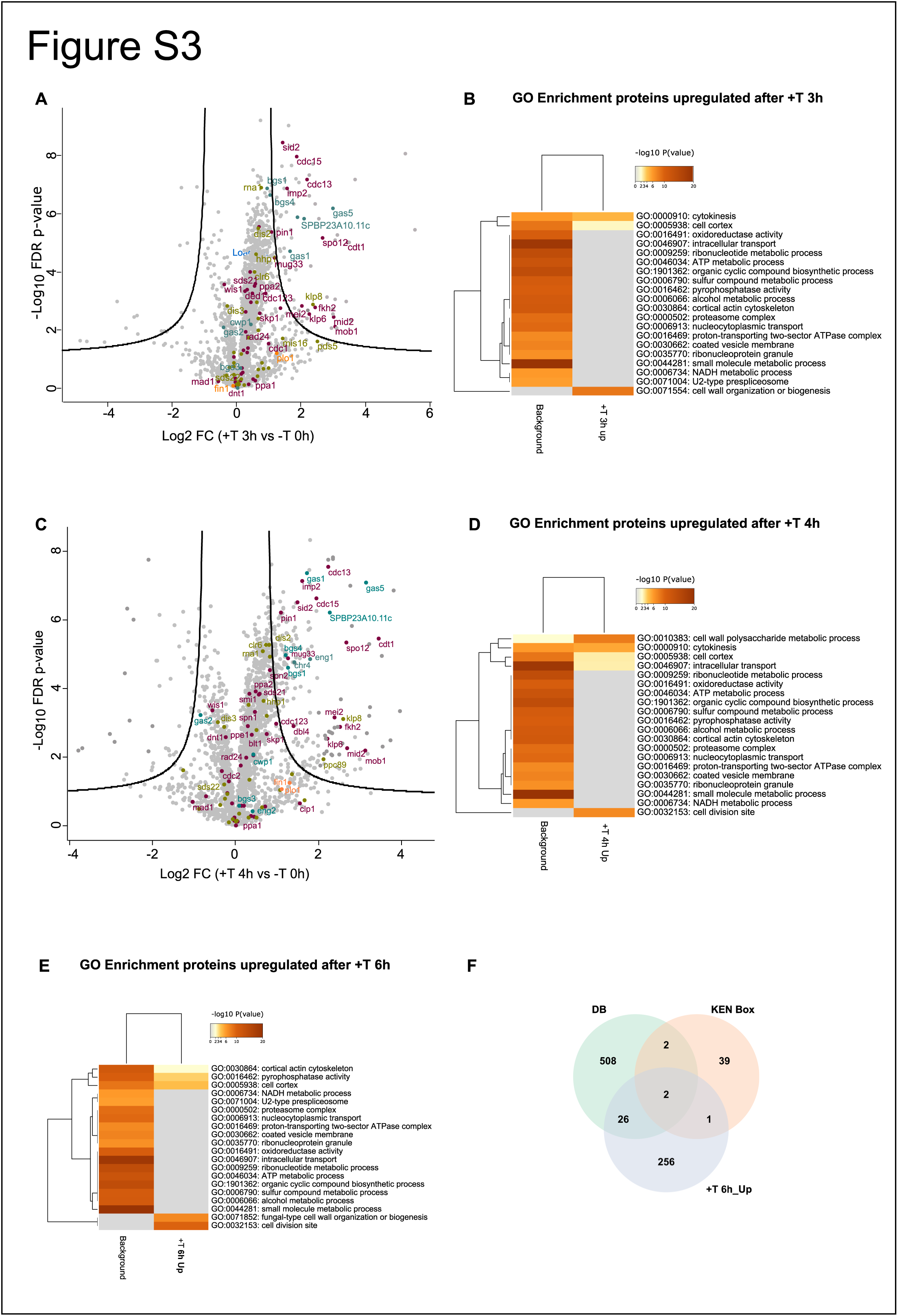

Supplement: Supplementary file 5 [file Image3.tif]

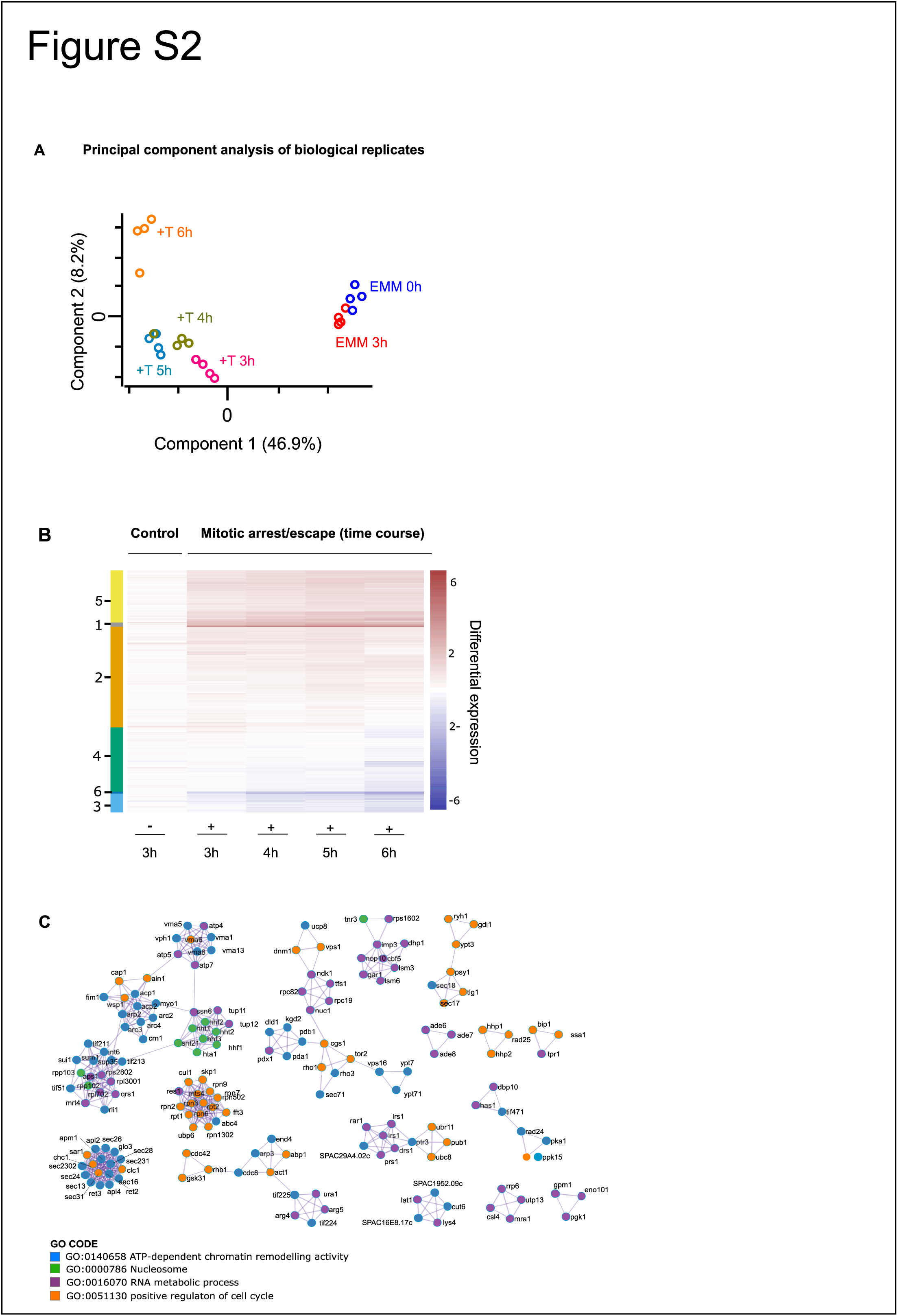

Supplement: Supplementary file 6 [file Image2.tif]
