# Supplementary material for: Uncoupling of Mitosis and Cytokinesis Upon a Prolonged Arrest in Metaphase Is Influenced by Protein Phosphatases and Mitotic Transcription in Fission Yeast
Source: Front Cell Dev Biol. 2022 Jul 18;10:876810. doi: 10.3389/fcell.2022.876810 (PMC9340479; doi:10.3389/fcell.2022.876810)
Supplement: Supplementary file 4 [file Presentation1.pdf]

SUPPLEMENTARY FIGURE LEGENDS

Supplementary Figure S1 (related to Fig.3).

(A) Overview of the simultaneous repression of *pab1* under the thiamine-regulated *p41nmt1* promoter and degradation of the protein under the mini version of the auxin-inducible degron (here referred to as *3PK-mAID-pab1* in the genetic background *Padh15-skp1-At-Tir1-2NLS-Padh15-skp1-Os-Tir1*). While thiamine leads to the repression of transcription, the addition of auxin leads to the recognition of 3PK-mAID-Pab1 by Tir1 and its subsequent ubiquitination and proteasome-mediated degradation.

(B) Representative images of DAPI and blankophor-stained ethanol-fixed cells of *p41nmt1slp1 41nmt1*-3PK-miniAID-pab1 under the presence of auxin but not thiamine (+A/-T). The same cells grown in EMM (-T/-A) were used as a control (left).

(C) FACS profile of *p41nmt1slp1* and *pnmt41slp1 par1Δ* ethanol-fixed cells stained with propidium iodide during a time course after thiamine addition. Wild type cells were used as a control (left).

Supplementary Figure S2 (related to Fig. 6).

(A) Principal component analysis (PCA) of the samples shows high reproducibility of replicates. The dimension 1 separates treatment and control and dimension 2 between time points. PCA was performed based on the proteins with significant differential expression determined by an ANOVA test using an FDR cut-off < 0.05 **(Table S3).**

(B) Heatmap of the differential expression levels between thiamine-treated samples vs the non-treated sample at 0 hours with a fold-change > 2-fold up or down (n= 1403). Red indicates up-regulated differential expression and blue indicates down-regulated expression at each indicated time point.

(C) PPI network of the significant proteins with changes in the dynamic of expression after *slp1* repression clustered in **A**. Proteins are clustered based on biological functions. Four clusters represented by one single GO-term that was chosen based on semantic similarity between multiple top-go terms from each cluster.

Supplementary Figure S3 (related to Fig. 6).

(A, C) Volcano plot. The log2 FC indicates the mean expression level for each protein. Each dot represents one protein. The line represents a cut-off of FDR < 0.01 with a

S0= 0.5. Beyond the line, black dots represent significant hits between EMM T0 and EMM +T 3 h **(A)** and +T 4 h **(C)**. Dots are coloured based on GO annotations: purple, cell cycle; green, chromosome segregation; orange, cytokinesis and septation and blue, 1-3 ß-glucanase activity.

(B, D, E) Top GO terms for the proteins upregulated after 3 h **(B)**, 4 h **(D)** and 6 h **(E)** of thiamine addition (See **Fig. 6E** for the related volcano plot). Enrichment analysis was performed on proteins with a differential expression >3x and an FDR adjusted p-values < 0.05. Metascape; All GO annotations, background gene list **(Supplementary Table S2)**, and a hypergeometric test with Benjamini–Hochberg correction. All GO terms are listed in **Supplementary Table S5**.

(F) Venn diagram. The numbers indicate the overlap between the list of significant proteins up-regulated at 6 h with thiamine and the proteins containing a Slp1 recognition motif (Destruction box (DB, RxxLxxxxN) and the KEN box (KENxxxN)). List of proteins that contain these motifs in their sequence were retrieved from Pombase (https://www.pombase.org/).
