# Supplementary material for: Uncoupling of Mitosis and Cytokinesis Upon a Prolonged Arrest in Metaphase Is Influenced by Protein Phosphatases and Mitotic Transcription in Fission Yeast
Source: Front Cell Dev Biol. 2022 Jul 18;10:876810. doi: 10.3389/fcell.2022.876810 (PMC9340479; doi:10.3389/fcell.2022.876810)
Supplement: Supplementary file 8 [file DataSheet1.PDF]

Table S1 matrix 40_R (ANOVA FDR 0.05, HC q-value (FDR-pvalue adjusted) 0.0001
Table S2 matrix 164 (105+ annotations)
Table S3 matrix 106 (ANOVA for PCA)
Table S4 GO enrichment for HC_PPI network GOTop3MCODE
Table S5 GO enrichment for Differential expression_PPI network UpT6 GOTop3MCODE
Table S6 Strains
Table S7 Oligos
